# Midfacial Morphology and Neandertal–Modern Human Interbreeding

**DOI:** 10.3390/biology11081163

**Published:** 2022-08-03

**Authors:** Steven E. Churchill, Kamryn Keys, Ann H. Ross

**Affiliations:** 1 Department of Evolutionary Anthropology, Duke University, Durham, NC 27708, USA; churchy@duke.edu; 2 Centre for the Exploration of the Deep Human Journey, University of the Witwatersrand, Johannesburg 2050, South Africa; 3 Human Identification & Forensic Analysis Laboratory, Department of Biological Sciences, North Carolina State University, Raleigh, NC 27695, USA; kkeys@ncsu.edu

**Keywords:** hybridization, introgression, ancient DNA (aDNA), hominin paleontology, paleoanthropology

## Abstract

**Simple Summary:**

Studies of human fossils, and the DNA extracted from them, reveal a complex history of interbreeding between various human lineages over the last one hundred thousand years. Of particular interest is the nature of the population interactions between the Neandertals of Ice Age Europe and western Asia and the modern humans that eventually replaced them. Here, we used six measurements of the facial skeleton, in samples of Neandertal and early modern human fossils, in an exploratory study aimed at trying to identify geographic regions (from the Near East to western Europe) where interbreeding may have been prevalent enough to have left a signal in the facial morphology of the early modern humans of those regions. Although fossil sample sizes were in some cases very small, the results are consistent with the Near East having played an important role in the introduction of Neandertal genes into the genomes of living humans.

**Abstract:**

Ancient DNA from, Neandertal and modern human fossils, and comparative morphological analyses of them, reveal a complex history of interbreeding between these lineages and the introgression of Neandertal genes into modern human genomes. Despite substantial increases in our knowledge of these events, the timing and geographic location of hybridization events remain unclear. Six measures of facial size and shape, from regional samples of Neandertals and early modern humans, were used in a multivariate exploratory analysis to try to identify regions in which early modern human facial morphology was more similar to that of Neandertals, which might thus represent regions of greater introgression of Neandertal genes. The results of canonical variates analysis and hierarchical cluster analysis suggest important affinities in facial morphology between both Middle and Upper Paleolithic early modern humans of the Near East with Neandertals, highlighting the importance of this region for interbreeding between the two lineages.

## 1. Introduction

The first two decades of the 21st Century brought a radical transformation to our understanding of the evolutionary history of the genus *Homo* during the Middle and Late Pleistocene. This metamorphosis has been driven by advances in ancient DNA (aDNA) and related studies, the increasing use of sophisticated, computationally-intensive morphometric analyses (such as 3D geometric morphometrics of the enamel–dentine junction of hominin teeth) and virtual imaging techniques, a continued emphasis on fieldwork and the recovery of new fossil specimens, and rigorous radiometric dating of important sites and specimens. In combination, these approaches have illuminated a complex picture of multiple, sympatric lineages of *Homo* persisting through the Pleistocene, with considerable interbreeding occurring between them. Highlights from this picture include: (1) the discovery of a previously unknown lineage (the Denisovans), apparently representing a sister species to the Neandertals, from an isolated fossil from 48–30 Ka-old layers in a Siberian cave [1]; (2) the recognition of persistent yet temporally and geographicallycomplex patterns of interbreeding between *Homo* lineages through the Middle and Late Pleistocene [2]; (3) the discovery of multiple, late-surviving, basal (based on their possession of ancestral morphological traits) species of *Homo* in Africa and Asia [3,4,5], one of which (*H. naledi*) may have been a source of introgressed archaic human genes in the modern African genome [6,7,8,9]; (4) virtual reconstruction, reanalysis, and dating of early *Homo sapiens* specimens that reveal an earlier origins of our species in Africa (at ca. 315 Ka) [10] and possibly an earlier incursion of modern humans into Europe (by ca. 210 Ka) [11,12] than previously known; and (5) the rediscovery of fossil material that has improved our understanding of the skeletal morphology of the Denisovans [13] and clarified the morphological and taxonomic affinities of Middle–Late Pleistocene Asian (possibly Denisovan) specimens [14].

While the paleontological and genomic records show interbreeding between *Homo* lineages to have been common across the Middle and Late Pleistocene Old World [6,15,16,17,18], much attention has been focused on the nature of introgression between the Neandertal and modern human lineages in Europe and western Asia. The picture that has emerged from aDNA and morphological studies suggests a divergence of the modern human and Neandertal/Denisovan lineages in the Middle Pleistocene (between 700–500 Ka) [19], separation of the Neandertal and Denisovan lineages shortly thereafter [20], and then repeated episodes of hybridization between the Neandertal and modern human lineages in both western Asia and Europe [2,21,22], as well as between the Denisovan and other lineages [17]. However, the nature of Neandertal–modern human hybridization remains unclear. Initial analysis of the Neandertal genome [23] noted similar amounts of Neandertal ancestry in recent humans from both Europe and Asia, suggesting that interbreeding had been limited to the Near East before 100 Ka (but see Sankararaman et al. [21] who estimate this date to be 65–47 Ka), during early range expansion of modern humans and prior to the divergence of living European and Asian populations. This scenario is also consistent with the nature of introgressed modern human genes in a ca. 50 Ka-old Neandertal from the Altai Mountains of Siberia [24]. Oddly, however, subsequent analyses of the Denisovan, Neandertal, and modern human genomes [25,26] revealed a greater amount of Neandertal ancestry in East Asian modern human populations than in those from Europe, indicating the introgression of Neandertal genes into the lineage leading to modern-day Asians after the separation of European and Asian lineages. The finding of a greater Neandertal contribution to East Asian than European populations is consistent with the complete absence of Neandertal mitochondrial DNA (mtDNA) in both early modern European fossils and living Europeans [27] and with the lack of evidence for gene flow from modern humans in the DNA of four late (<45 Ka) Neandertals from Belgium, France, and western Russia [28]. That modern-day Europeans do not carry a greater percentage of Neandertal nuclear DNA than Asians is surprising, given the potentially longer period of overlap between Neandertals and early modern humans in Europe than elsewhere [29], and thus greater opportunity for interbreeding, and also given morphological evidence (reviewed below) of admixture in the European fossil record. Nonetheless, Neandertal genes do not appear to be apportioned equally in living Europeans and Asians [30]. One geographic region in which Neandertal DNA may have uniquely introgressed into Asian but not European lineages of modern humans is on the eastern edge of the Neandertal range: the genome of a 45 Ka-old modern human from Ust’-Ishim in Siberia documents Neandertal introgression consistent with a hybridization event some 13–7 Ka earlier [31]. Hybridization in western Asia may constitute a “second pulse” of introgression after initial contact in the Near East, as suggested by some analyses of the genomic data [32]. Continued eastward expansion of modern human populations after hybridizing with Neandertals on the easternmost edge of their distribution could account for living Asians carrying, on average, a higher percentage of Neandertal ancestry than do living Europeans.

**Figure 1:**
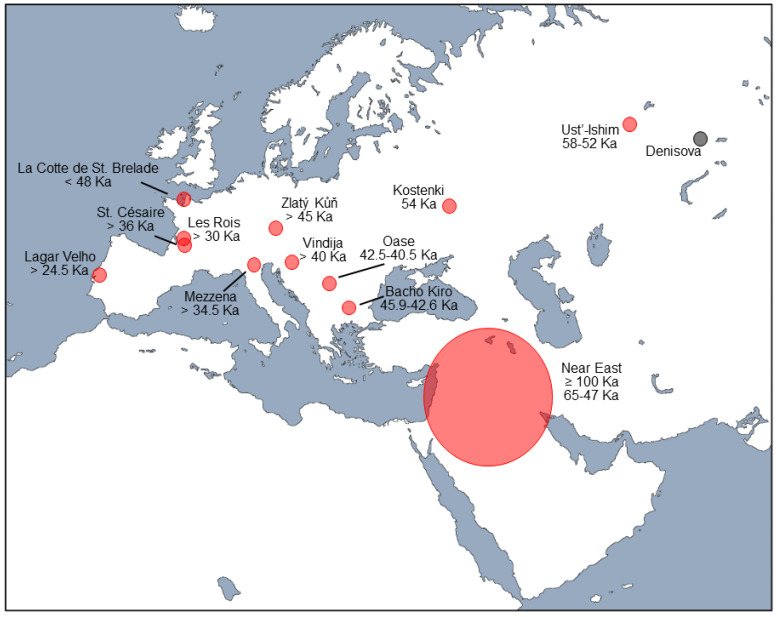
Map of western Eurasia showing areas and estimated dates of possible Neandertal–modern human hybridization (in red) based on fossil samples from indicated sites. Ancient DNA from a Neandertal fossil from Denisova Cave (black dot) has been interpreted as reflecting Neandertal–modern human admixture in the Near East at 100 Ka or earlier. See text for details.

However, more recent morphometric and aDNA studies, focused primarily on early modern European fossils, have produced compelling evidence of Neandertal–modern human hybridization in Europe (Figure 1). Ancient DNA from Kostenki 14, a 38.7–36.2 Ka-old modern human from the western portion of the Russian Plain, contains longer segments of Neandertal DNA than are found in living Europeans, producing an estimated hybridization date of ca. 54 Ka [33]. Unlike the somewhat older specimen from farther east at Ust’-Ishim [31], the Kostenki individual is genetically closer to present-day Europeans than East Asians [33]. Likewise, a relatively high proportion (6–9%) of the genome of a 42–37 Ka-old modern human from Romania, Oase 1, appears to have derived from Neandertals, consistent with this individual having had a Neandertal ancestor some four-to-six generations earlier [34]. This finding confirmed earlier suggestions, based on the presence of derived Neandertal features in the skull and postcranial skeleton, that the fossils from the Peştera cu Oase, as well as those of nearby Muierii, showed morphological evidence of Neandertal admixture [35,36,37] (but see [38]). A similarly recent hybridization event (six or seven generations earlier) has been inferred from aDNA of early modern humans at Bacho Kiro, Bulgaria, around 45.9–42.6 Ka [39], while a somewhat more distant event (70–80 generations earlier) has been recognized in the genome of a >45 Ka-old cranium from Zlatý kůň in Czechia [40]. Note, however, that multivariate analysis of cranial metrics of comparably-aged crania from the Czechian site of Mladeč failed to detect a morphological signal of admixture [41]. Despite the apparent lack of such a signal, the aDNA evidence unequivocally indicates hybridization events in Europe. This genetic evidence, however, derives entirely from sites in central and eastern Europe, and thus the extent (if any) of interbreeding that occurred between Neandertals and modern humans in western Europe remains unclear.

However, morphological indicators of interbreeding have long been recognized in the fossil record of Late Pleistocene Europe (including western Europe), in the form of both the persistence of derived Neandertal features in European early modern humans [35,37,42,43,44], and in the existence of possible hybrid or near-hybrid individuals [45,46,47]. For example, traditional and morphometric comparisons of a sample of teeth from Middle Paleolithic deposits on the south coast of Jersey (Channel Islands) may provide morphological evidence of Neandertal–modern human hybridization in the most western reaches of Europe [48]. These teeth, which postdate 48 Ka, evince a mix of Neandertal and modern human traits and tend to fall between Neandertal and recent human samples in morphometric shape space, leading Compton et al. [48] to suggest that they may derive from a hybrid population. These authors [48] also suggest that the morphologically intermediate Neandertal teeth from 45–38 Ka-old sediments at Palomas (Spain) [49] may likewise reflect an admixed population.

Of interest, too, is the presence of Neandertal features in early- and mid-Upper Paleolithic modern human fossils from France (at Les Rois [47]) and Portugal (at Lagar Velho [46]), which, when combined with a similar finding in the fossils from the Channel Islands [48] discussed above, may reflect an appreciable hybridization zone in western Europe. In addition, a number of relatively late-surviving Neandertals in western, southern, and central Europe have been argued to evince a greater degree of modern human morphology than their older conspecifics, suggesting gene flow from invading modern humans into the resident Neandertal populations across most of Europe. These somewhat more modern-looking Neandertals have been identified at St. Cesaire, France [50,51], Vindija, Croatia [52,53,54,55], and Riparo Mezzena, Italy [45].

Efforts to interpret the morphological evidence are hampered by unclear expectations of how admixture is expressed in skeletal morphology (see [38]). While much of the literature has focused on the persistence of Neandertal autapomorphies in early modern European fossils, a high frequency of dental and sutural anomalies may also provide a skeletal signal of hybridization in a population [56]. In this regard, Ackermann [56] pointed to a high frequency (36%) of rotated mandibular premolars in the Krapina (Croatia) Neandertal sample as a potential indicator of admixture. At ca. 130 Ka [57], the Krapina Neandertals antedate the widespread incursion of modern humans into Europe (which may have begun as early as 48 Ka [29]). Thus, if they do represent an admixed population, then either some populations of central European Neandertals must have interbred with earlier waves of modern human migration into the Balkans (as possibly represented by the ca. 210 Ka-old modern human from Apidima, Greece [11]; note however that the Apidima 1 cranium is not universally accepted as representing a modern human [58]), or they must represent the introgression of modern human genes via gene flow from a hybrid zone in the Near East (established perhaps as early as 194 Ka [12]), in advance of the actual in-migration of modern humans. This latter mechanism has been invoked to explain the presence of some modern human-like features in the morphology of the ca. 40 Ka-old Neandertals from the G_3_ layer at the nearby Croatian site of Vindija [52]. Thus, the morphological evidence may be documenting a richer history of Neandertal–modern human admixture in Europe than seen in the current aDNA record, but one that was undoubtedly complex [32].

To further explore the potential morphological signal of Neandertal–modern human admixture, we here assess the degree of morphometric similarity in the facial skeletons of Neandertals and early modern humans across seven geographic regions: the Near East, and eastern, central, southeastern (Balkans), western, southwestern (Iberian and Apennine peninsulas and Mediterranean France), and northern Europe. We focus on the facial skeleton because it has been shown to be a good indicator of population affinity [59], because it may reflect those affinities more faithfully than the cranial vault in Pleistocene humans [60], because Neandertals are characterized by distinctive facial morphology [61], and because at least one early modern European sample (Muierii [35]) has been argued to express Neandertal-like aspects in its facial form. While we expect, a priori, all modern human groups to be more similar to one another than any are to the Neandertals [62], we might also expect—if there was regional variation in levels of hybridization—that the ways that early modern European groups differ from one another in facial morphology might reflect variation in the Neandertal contributions to their genomes.

## 2. Materials and Methods

### 2.1. Samples and Measurements

Facial metric data were obtained from the literature [63,64,65,66,67,68] for 12 fossil and recent human samples, totaling 316 specimens (see Supplementary Materials). Neandertals (*n* = 13) were subdivided into Near Eastern (NE NEAN; *n* = 5), southern European (SE NEAN; *n* = 5), and western European (WE NEAN; *n* = 3) samples, reflecting geographic regions known to have some genetic separation [69] (but note that sample size considerations necessitated combining the Neandertals from southeastern and southwestern Europe into a single southern European sample). Data from anatomically modern human fossils (AMH, *n* = 201), from Upper Paleolithic and Mesolithic contexts across Europe and the Near East, were also subdivided by geographic region: Near Eastern/North African (NE/NA AMH; *n* = 3), and eastern (EE AMH; *n* = 24), southeastern (SE AMH; *n* = 23), central (CE AMH; *n* = 49), northern (NE AMH; *n* = 25), southwestern (SW AMH; *n* = 35), and western (WE AMH; *n* = 42) European samples. Temporal representation across the samples is not uniform: the NE/NA AMH is an Upper Paleolithic sample, the NE AMH is a Mesolithic sample, and the others are combined Upper Paleolithic/Mesolithic samples (with two of them, SE AMH and EE AMH, being largely Mesolithic) (see Supplementary Materials for details of sample composition). Since Neandertal–modern human hybridization has been hypothesized to have occurred in the Near East at potentially early (>100 Ka) and later (65–47 Ka) dates (see above), we also included a sample of Near Eastern, Middle Paleolithic-associated fossils (NE MP AMH; *n* = 5), from the sites of Skhūl and Qafzeh (Israel), with radiometric dates ≥ 90 Ka. We also included data from a recent human sample from east Africa (EA RECENT; *n* = 83) as an outgroup (that is, a group not expected to have significant Neandertal ancestry).

**Figure 2:**
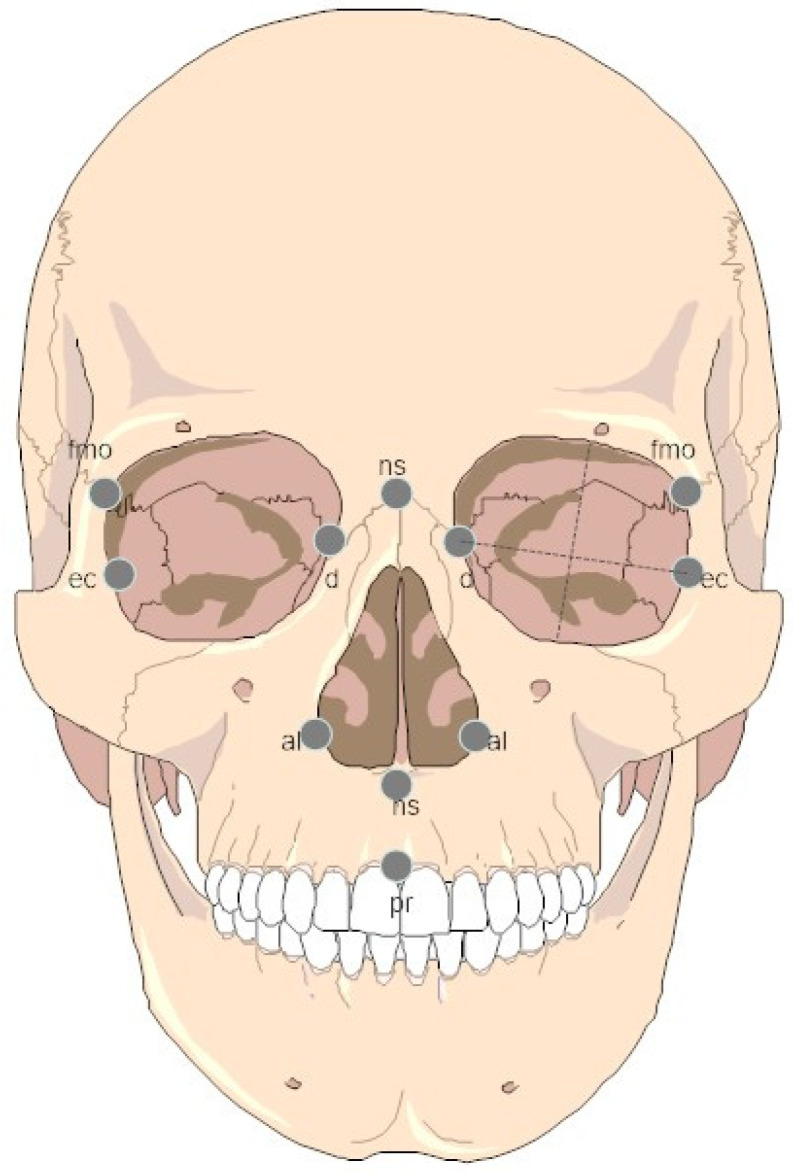
Facial landmark locations for the measurements used.

The selection of traditional craniometric measurements was based on the consistency and availability of measurements from different sources, which limited the study to six measurements (Table 1, Figure 2). An additional consideration was that these measurements are part of the standard forensic set that has been tested for reliability and repeatability, allowing for the incorporation of data from several sources with minimal inter-observer error [70,71,72]. Furthermore, midfacial measures have been found to be the most morphologically informative when examining craniofacial variation in contemporary contexts, as they have been linked to climatic adaptations (which may differentiate regional groups), and because they are minimally affected by developmentally plastic alterations of the cranial vault [73,74]. Craniofacial measurements used in this study are listed in Table 1 and the landmarks upon which they are based are illustrated in Figure 2. To include as many specimens as possible, it was necessary to impute missing variables in some individuals. However, individuals were not included in this analysis if they were missing more than two variables (≤33%). Because sample sizes were small within regions, simple mean substitution was used to estimate the missing values. Sex variation is negligible within each group included in population studies and thus, males and females were pooled to incorporate all of the observed biological information and to increase sample sizes [75]. To examine the effect of climate on facial morphology, each sample was scored and numerically coded using the climate map for biodiversity [76]. The recent human samples were scored according to Metzger [76], while fossil samples were scored using paleoclimatic data [77,78,79]. Radiocarbon dates associated with each fossil specimen (see Supplementary Materials) were taken from the literature [63,64,65,66,67,68] and were used to evaluate the effect of time on facial morphology across our samples.

Because significant facial size differences have been shown between *Homo* groups [62], variables were size adjusted following Darroch and Mosimann [80,81], without log transformation. Size and shape variables were calculated from the raw measurements, where size is defined as the geometric mean (GM) of all six variables. The GM of the six variables was calculated as follows:

(1) $$Size=(\prod_{i=1}\;X_{i}{)}^{1/n}$$

and the raw variables were divided by the GM to create new shape variables (*Y = X/SIZE*, where *X* is the raw measurement). While these newly created shape variables do not remove absolute size (only geometric morphometric approaches truly remove size), they are scale-free and provide a better understanding of the geometric or shape-related similarity among the groups [81].

### 2.2. Multivariate Statistics

A canonical variates analysis (CVA: a linear combination of predictor variables that summarize among-population variation) was conducted using the newly calculated shape variables [82]. Among-group differentiation was measured using Mahalanobis squared distances, which is a similarity measure and a function of group means and the pooled variance–covariance matrix [82]. A hierarchical (or agglomerative) cluster analysis using average linkage was conducted on the Mahalanobis squared distances to examine group similarity [70]. All statistical analyses were conducted in SAS 9.4 [83] and the hierarchical cluster analysis was conducted in JMP 16 Pro [84].

### 2.3. Spatial Analyses

A Pearson’s product–moment correlation coefficient was performed to assess the relationship between climatic zones, geometric mean (i.e., size), and the shape variables. Further, a Pearson’s product–moment correlation was used to assess size and shape changes over time. Dutilleul’s [85] estimator was used to correct for spatial autocorrelations and was performed using PASSaGE: Pattern Analysis, Spatial Statistics and Geographic Exegesis Version 2 [86].

## 3. Results

Table 2 presents the group means for the raw variables.

### 3.1. Multivariate Statistics

The Mahalanobis squared distances are presented in Table 3. No significant differences were detected between SE AMH and WE AMH, or between NE and SW AMH samples. Likewise, the western European Neandertal sample is not significantly different from either the Near Eastern or southern European Neandertal samples. While all the other groups are significantly different from one another, all the AMH samples are fairly similar given the small distance values. The Near Eastern Middle Paleolithic AMH sample is closest to the southwestern European AMH sample. Furthermore, the East African recent sample is similar to various AMH groups. Interestingly, the Near Eastern Neandertal sample is most dissimilar to the Near Eastern North Africa AMH sample.

**Figure 3:**
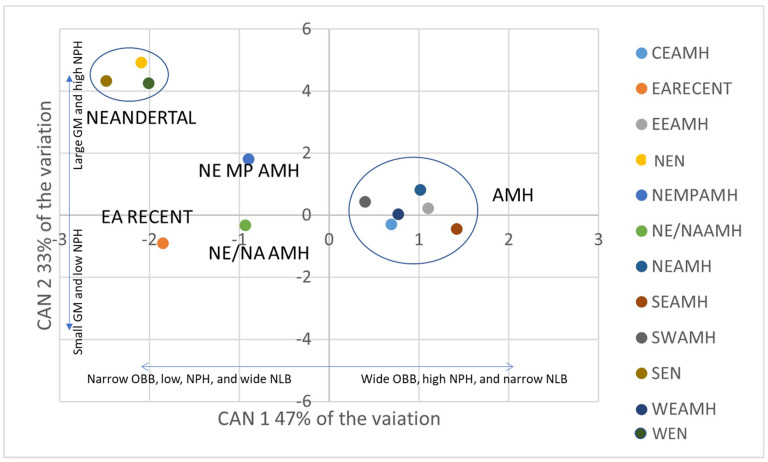
A plot of CVA canonical axis 2 (CAN 2) on axis 1 (CAN 1) represents 80% of the total variation. Neandertals cluster together (narrow orbits, wide nasal breadth, and large geometric mean); Near Eastern Middle Paleolithic AMH fall in between the Neandertal and the other AMH samples. The Near Eastern/North African AMH sample has intermediate orbit and nasal breadths, and a small geometric mean and is closest to the recent East African sample.

Table 4 presents the significant canonical roots, with 80 percent of the variation depicted on the first two canonical variates (CAN 1 and CAN 2). The canonical structure (Table 5) shows that the variation exhibited on CAN 1 is related to orbital, nasal breadth, and a moderate degree to NPH. At the same time, that on the second axis (CAN 2) is related to size (geometric mean), and moderately to NPH. The plot of CAN 1 and CAN 2 (Figure 3) shows that Neandertals have narrow orbits, wide nasal breadths, and large faces overall. The European AMH have wider orbits, narrower nasal breadths, and smaller geometric means. The Near Eastern Middle Paleolithic AMH sample has an orbital and nasal breadth and geometric mean values that are intermediate between the Neandertal and European AMH samples. The Near Eastern/North African AMH sample has intermediate orbital and nasal breadth, combined with a small geometric mean. The East African recent sample has narrow orbits, wide nasal breadth, and the smallest geometric mean.

**Figure 4:**
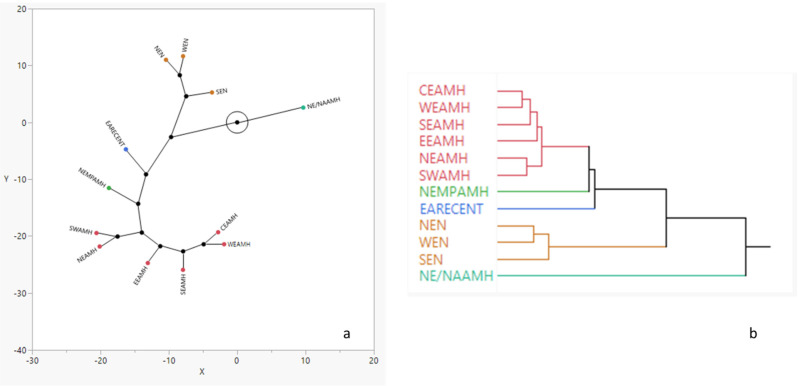
Constellation plot (**a**) and dendrogram (**b**) results from hierarchical cluster analysis showing group relationships.

The hierarchical cluster analysis using the Mahalanobis squared distances shows two distinct clusters: (1) anatomically modern humans (fossil and recent), and (2) Neandertals (Figure 4a). All of the European fossil AMH groups cluster together. The Near Eastern Middle Paleolithic sample leaf branches off this European AMH cluster, but is significantly dissimilar. Surprisingly, the East African recent human sample branches off of the stem leading to the European AMHs and the NE MP AMHs (Figure 4a). Among the modern human samples, the most dissimilar is the Near Eastern/North African AMH series (Figure 4a).

The constellation plot, which arranges the groups as endpoints, further illustrates the similarity/dissimilarity of the groups (Figure 4b). The morphological distance between cluster joints is illustrated by the length of the line between them. The constellation plot shows that the most distinct group is the Near Eastern/North African AMH, which is no closer to the other modern human samples than it is to Neandertals. With this one exception, all of the anatomically modern human samples are closer to one another than they are to the Neandertal samples.

### 3.2. Spatial Analysis

The Pearson’s product-moment correlation using the Ditulleul [85] method to correct for spatial autocorrelation revealed no significant correlation between the climatic zones and shape variables (NPHs, r = −0.523, *p*-value = 0.234; FMBs, r = 0.132, *p*-value = 0.307; NLHs, r = −0.244, *p*- value = 0.383; NLBs, r = 0.468, *p*-value = 0.303; OBBs, r = −0.365, *p*-value = 0.314; OBHs, r = 0.303, *p*-value = 0.336), nor between climatic zones and geometric means (r = −0.162; *p*-value = 0.340), indicating that climate does not model either craniofacial shape or size (at least in these samples). The only significant variable correlated with temporality was the geometric mean, our measure of size (r = 0.56, *p*-value > 0.001; NPHs, r = 0.23, *p*-value = 0.088; FMBs, r = −0.137, *p*-value = 0.061; NLHs, r = 0.016, *p*-value = 0.882; NLBs, r = 0.184, *p*-value = 0.178; OBBs, r = −0.184, *p*-value = 0.112; OBHs, r = −0.171, *p*-value = 0.106).

## 4. Discussion

Neandertals possessed distinctive facial morphology, characterized by large, superoinferiorly tall yet mediolaterally narrow faces with pronounced midfacial prognathism, absolutely and relatively tall orbits, flat or convex infraorbital plates (and thus, no canine fossae), wide nasal apertures and strongly projecting external noses, and prominent, double-arched supraorbital tori [87,88,89]. This constellation of features is, unfortunately, not perfectly represented by the measures of overall facial shape used in this analysis (for example, the inclusion of a measure of facial length might be expected to have high utility in distinguishing Neandertal from modern groups [90]). Nonetheless, the variables used here should be expected to reasonably represent overall facial size and shape (that is, relatively wide versus relatively narrow faces), the shape of the orbits (relative orbital width), and the shape of the nasal aperture (relative nasal width). The large size and distinctive morphology of the Neandertal face does indeed appear to be captured on the first two canonical axes of the CVA (Figure 2), which in combination separate the Neandertal samples from the fossil and recent modern human samples. The strong negative values of the Neandertal samples on CAN 1 reflect faces with wide nasal apertures and narrow orbits (relative to overall facial size), while their strong positive values on CAN 2 denote large faces with elevated upper facial heights (relative to overall facial size). Note that upper facial height (NPH) has moderate yet positive loadings on both CAN 1 and 2 (Table 5), which we interpret as reflecting, on CAN 1, the utility of this variable in differentiating modern human groups, while CAN 2 accounts for residual variation in NPH (that is, variation not explained on CAN 1) that serves (in part) to separate Neandertal from modern human samples. All of the anatomically modern European groups have positive values on canonical axis 1 and largely neutral values on axis 2, reflecting smaller faces overall, with relatively narrower noses and wider orbits.

At the risk of over-interpreting the position of very small fossil samples, we find the intermediate positions (between Neandertal and European AMH samples) of the Near Eastern Middle Paleolithic AMH and the Near Eastern/North African AMH samples to be interesting, as this might reflect a greater contribution of Neandertal genes to facial morphology in the Near East. Relative to European fossil modern humans, the NE MP AMH sample exhibits a more Neandertal-like constellation of facial size and morphological features (Figure 3). Given the antiquity of this sample, it is possible that these fossils represent a population that had not yet fully evolved the derived modern human condition of smaller faces, and which may retain (to some degree) ancestral features such as wide nasal apertures (which in turn may be related to the plesiomorphic retention of facial prognathism [90,91]). However, the modern human pattern is already evident in the facial morphology of two specimens from Jebel Irhoud (Morocco) [10], which considerably antedate the fossils from Skhūl and Qafzeh. Interestingly, the fossils from Irhoud, as well as from Skhūl and Qafzeh, fall within the range of variation of recent modern humans—and distinct from Neandertals—in a principal components analysis (PCA) of facial shape metrics [10]. However, Qafzeh 9 falls on the edge of the recent human distribution, and in the direction of Neandertals in shape space, as does (but to a lesser extent) Skhūl 5 (Figure 4a in [10]). Qafzeh 6, on the other hand, falls well away from the Neandertals. Note, however, that this PCA was conducted on size-scaled 3D geometric morphometric shape variables [10], and thus does not factor in variation within and between groups in facial size. Overall, the intermediate position of the NE MP AMH sample between the Neandertal and other fossil AMH samples (in our analysis) could be interpreted as reflecting sufficient Neandertal–modern human hybridization in the Near East, at a sufficiently early date (>100 Ka), to affect the facial morphology of the early modern human fossils from Skhūl and Qafzeh.

It is also interesting to note that the NE/NA AMH sample appears (based on its position on CAN 1) to be similar to the NE MP AMH sample in facial shape but to have a facial size (based on its position on CAN 2) that falls among most other fossil and recent modern human samples. Again, at the risk of over-interpreting morphology in a very small fossil sample, this may be a signal of Neandertal introgression in the Near East (but perhaps later in time, consistent with the inference of hybridization there between 65–47 Ka [21]). The east African recent human sample falls with Neandertals on CAN 1, and with most of the other modern human groups on CAN 2 (Figure 3). This sample, representing the Teita from Kenya [71], has mean nasal breadths (NLB; Table 2) that fall at the high end of the range of sample means, likely representing climatic adaptation to the hot and humid climate of southeastern Kenya. We thus interpret their position on canonical axis 1 to reflect convergence in nasal morphology, rather than homology.

Hierarchical clusters based on Mahalanobis squared distances produce an interesting picture (Figure 4). Under the (arguably false) assumption that the facial metrics employed in this study are selectively neutral (i.e., following neutral microevolutionary processes [59]) and thus accurately reflecting relationships between populations), and assuming no hybridization whatsoever between groups, we would expect clusters that show two distinct clades (Neandertals versus modern humans), and clear geographic (or possibly temporal plus geographic) structure within each clade. Expectations given hybridization—particularly involving temporally complex and potentially small-scale hybridization events, distributed across geographic space—are unclear. While the cluster analysis did largely produce separate Neandertal and modern human clades, the Neandertal clade is nested within the modern human cluster (with the Near Eastern/North African AMH sample being the sole outgroup). With the exception of this outgroup, the plots show good (but not perfect) geographic structure: on the AMH branch, all European AMHs cluster together, joined by the NE MP AMH sample, which is then joined by the EA RECENT sample. Within the European AMH cluster, however, geographic structure breaks down (for example, northern and southwestern early modern Europeans cluster together). Likewise, the Neandertal clade does not show the expected geographic structure, in which western and southern European Neandertals should be closer to one another than either is to Near Eastern Neandertals. Complexity in the hierarchical clustering of both the AMH and Neandertal samples is no doubt due to small fossil sample sizes, variation in the average geological age of the AMH samples (although we find that only overall facial size significantly varies with time), the effects of natural selection on aspects of facial morphology, and the imperfect way in which facial morphology reflects populational relationships. Still, we find it interesting that, in multivariate shape space, the Near Eastern/North African AMH sample is equidistant from the Neandertal and all other modern human samples. This result is consistent with a significant Neandertal signal in the Upper Paleolithic peoples of the Near East and northeastern Africa, and thus consistent with evidence suggesting that the Near East was a locus of hybridization on the order of 65–47 Ka [21]. While molecular and morphological evidence (reviewed above) clearly indicates some level of interbreeding between Neandertals and modern humans across the entire Neandertal range, such interbreeding is not clearly reflected in the limited analysis performed here.

The spatial analysis failed to detect significant relationships between any aspect of facial size or shape and climatic zones, which is surprising given the large body of literature that documents such relationships across global samples of modern humans (see [73,88]). In particular, we would expect both overall facial size and nasal size to covary inversely with temperature, and nasal breadth to covary positively with precipitation and temperature [73]. It is likely that two factors, in combination, obscured a climatic signal in the data. First, the bulk of the data represents populations from a somewhat limited geographic area (relative to the global samples that are usually employed to assess ecogeographic variation). These data also primarily represents Mesolithic populations, who may not have experienced the colder climatic extremes that are likely to affect facial morphology [73]. Second, Neandertals retained the plesiomorphic condition of wide noses [88], despite inhabiting cold–temperate environments. Thus, our samples included two groups with absolutely and relatively wide noses—the Neandertals and the Kenyan Teita—who lived in the climatic extremes of cold/dry and hot/wet environments, respectively. Given the relative primacy of nasal morphology in human adaptation to climate, the combination of these groups with similar nasal morphology yet very different climatic zones is likely to have weakened the morphological signal of climatic variation but may also reflect the complex and multifactorial mechanisms that shape craniofacial morphology [92].

Again, we caution that these results are based on very small sample sizes, and any interpretation of them should be viewed with caution. While this analysis was both exploratory in nature and limited to facial metrics available from the literature, the results suggest that there may be utility in expanding this approach, both by including metrics that may capture additional aspects of Neandertal facial morphology (such as midfacial prognathism) and by augmenting sample sizes to the extent possible (e.g., incorporating data from Natufian specimens in the NA/NE AMH sample).

## 5. Conclusions

This exploratory, multivariate analysis incorporated only six variables, which reflect only the size and shape of the face overall, orbital shape, and nasal aperture shape. Furthermore, the analysis was conducted on samples that, when temporally and taxonomically constrained, were woefully small, or, when of adequate sample size, represented populations (namely, Mesolithic peoples) not temporally close to potential Neandertal–modern human hybridization events. Despite these limitations, the separation of Neandertals from all modern humans in the multivariate space created by the first two canonical axes of the CVA, combined with the hierarchical clustering of distinct Neandertal and European AMH clades when Mahalanobis distances are considered, shows the utility of analyzing facial morphology for the information it may contain about population relationships and potential Neandertal–modern human interbreeding. Two samples, one representing Middle Paleolithic-associated early modern humans from the Near East, the other representing Near Eastern and northeast African Upper Paleolithic modern humans, were found to be either intermediate between the Neandertals and all other modern human samples in multivariate space (both samples, but especially the former), or to form a hierarchical branch in the cluster analysis unlike any other modern human sample (the later sample). While caution should be used in interpreting the results of analyses based on small sample sizes, these results could be considered consistent with the Near East being a substantial locus of Neandertal–modern human hybridization. This in no way negates the abundant aDNA and morphological evidence that suggests that such hybridization occurred across all (except perhaps northern) Europe, but simply that interbreeding in Europe was of a nature that it did not leave a clear and interpretable signal (at least given the limitation of the current study) in the facial morphology of most Late Pleistocene, early Holocene European modern humans.

## Figures and Tables

**Table 1:** Traditional craniometric measurements utilized in this study.

Measurement	Abbreviation	Description
Upper Facial Height (n-pr)	NPH	[71]
Bifrontal Breadth (fmo-fmo)	FMB	[71]
Nasal Height (n-ns)	NLH	[71]
Nasal Breadth (al-al)	NLB	[71]
Orbital Height	OBH	[71]
Orbital Breadth (d-ec)	OBB	[71]

**Table 2:** Group means for facial variables (standard deviations in parentheses).

	CE AMH	EA RECENT	EE AMH	NE MP AMH	NE NEAN	NE/NA AMH	NE AMH	SE AMH	SW AMH	SE NEAN	WE AMH	WE NEAN
NPH	67 (5.21)	63 (4.8)	70 (5.28)	75 (2.7)	88 (6.06)	65 (4.16)	72 (4.45)	67 (4.15)	70 (4.6)	88 (14.14)	68 (5.2)	85 (4.9)
FMB	96 (4.87)	97 4.04)	96 (5.32)	103 (5.54)	114 (4.18)	103.5 (3.5)	101 (4.62)	94 (5.05)	99 (5.01)	111 (10.52)	98 (4.56)	113 (1.2)
NLH	50 (4.7)	48 (3.55)	52 (3.5)	54 (1.3)	63 (3.9)	46 (1.76)	52 (3.19)	49 (3.16)	52 (3.71)	59 (4.28)	50 (4.09)	60 (0.6)
NLB	25 (2.21)	28 (1.87)	25 (2.58)	30 (1.41)	34 (2.88)	26 (1.54)	24 (2.06)	24 (2.05)	25 (1.9)	34 (4.3)	25 (2.17)	32 (3.8)
OBB	42 (3.04)	39 (1.76)	43 (2.54)	44 (1.79)	46 (1.24)	42 (3.8)	43 (1.73	42 (.67)	42 (2.56)	44 (1.22)	41 (2.9)	44 (2.1)
OBH	30 (2.41)	33 (1.9)	32 (2.25)	33 (3.39)	36 (0.89)	28 (1.53)	32 (2.46)	30 (2.5)	33 (3.31)	37 (1.52)	30 (2.0)	37 (1.2)

**Table 3:** Mahalanobis squared distances.

	CEA AMH	EA RECENT	EE AMH	NE MP AMH	NE NEAN	NE/NA AMH	NE AMH	SE AMH	SW AMH	SE NEAN	WE AMH
**EA RECENT**	7.15	0									
**EE AMH**	1.22	10.63	0								
**NE MP AMH**	7.78	9.62	7.96	0							
**NE NEAN**	35.19	34.54	32.99	11.88	0						
**NE/NA AMH**	51.76	52.84	57.27	59.71	81.24	0					
**NE AMH**	2.59	11.59	1.99	7.64	28.19	54.78	0				
**SE AMH**	0.89	11.13	1.51	11.56	42.00	55.13	2.57	0			
**SWAMH**	1.70	7.15	1.22	6.08	27.66	54.25	0.82 *	2.60	0		
**SE NEAN**	32.87	29.27	32.20	11.27	2.99 *	69.72	26.82	38.68	25.92	0	
**WE AMH**	0.56 *	8.04	1.81	7.81	32.83	52.40	1.15	1.14	1.18	30.58	0
**WE NEAN**	28.71	26.83	27.15	9.25	1.28 *	74.71	21.29	34.40	20.87	2.03 *	25.76

* not significantly different. All other groups are significantly different from each at >0.03 level.

**Table 4:** Significant canonical axis for the shape transformed variables.

No.	Eigenvalue	Cumulative %	Proportion	Likelihood Ratio	Approximate F	Num DF	Den DF	Pr > F
1	1.80	0.47	0.47	0.08	12.17	77	1793	<0.0001
2	1.28	0.80	0.33	0.23	8.62	60	1572	<0.0001
3	0.51	0.96	0.03	0.78	4.79	45	1111	<0.0001
4	0.12	0.98	0.03	0.70	2.48	32	1542	<0.0001
5	0.09	0.99	0.02	0.87	2.04	21	868	0.004

**Table 5:** Total canonical structure for the shape transformed variables.

Variable	CAN 1	CAN 2	CAN 3	CAN 4	CAN 5
GM	−0.27	0.93	−0.08	0.05	−0.07
NPHs	0.50	0.60	0.15	0.04	0.06
FMBs	−0.06	−0.25	0.31	−0.56	0.39
NLHs	0.38	0.25	−0.04	0.12	−0.13
NLBs	−0.81	−0.11	0.12	0.29	0.11
OBBs	0.71	−0.12	0.07	0.07	−0.07
OBHs	−0.37	−0.45	−0.28	−0.31	−0.43

## Data Availability

The data presented in this study are available in the Supplementary Material.

## Supplementary Materials

The following supporting information can be downloaded at: https://www.mdpi.com/article/10.3390/biology11081163/s1, Sample provenience, climate zone scores, and raw data.
