# Supplementary material for: Midfacial Morphology and Neandertal–Modern Human Interbreeding
Source: Biology (Basel). 2022 Aug 3;11(8):1163. doi: 10.3390/biology11081163 (PMC9404802; doi:10.3390/biology11081163)
Supplement: Supplementary file 1 [file biology-11-01163-s001.zip › biology-1825896-supplementary.pdf]

Deme	ID	**Deme (Region)**	**Country**
NE NEANDEF	MEN1	Near Eastern Neanderthals	Israel
NE NEANDEF	MEN2	Near Eastern Neanderthals	Iraq
NE NEANDEF	MEN3	Near Eastern Neanderthals	Iraq
NE NEANDEF	MEN4	Near Eastern Neanderthals	Iraq
NE NEANDEF	MEN5	Near Eastern Neanderthals	Israel
NEMPAMH	MEMPAMH2	Near Eastern MP AMH	Israel
NEMPAMH	MEMPAMH3	Near Eastern MP AMH	Israel
NEMPAMH	MEMPAMH4	Near Eastern MP AMH	Israel
NEMPAMH	MEMPAMH5	Near Eastern MP AMH	Israel
NEMPAMH	MEMPAMH6	Near Eastern MP AMH	Israel
SEAMH	SEAMH1	Southeastern European AMH	Greece
SEAMH	SEAMH2	Southeastern European AMH	Greece
SEAMH	SEAMH3	Southeastern European AMH	Romania
SEAMH	SEAMH4	Southeastern European AMH	Serbia
SEAMH	SEAMH5	Southeastern European AMH	Greece
SEAMH	SEAMH6	Southeastern European AMH	Serbia
SEAMH	SEAMH7	Southeastern European AMH	Serbia
SEAMH	SEAMH8	Southeastern European AMH	Serbia
SEAMH	SEAMH9	Southeastern European AMH	Serbia
SEAMH	SEAMH10	Southeastern European AMH	Serbia
SEAMH	SEAMH11	Southeastern European AMH	Serbia
SEAMH	SEAMH12	Southeastern European AMH	Serbia
SEAMH	SEAMH13	Southeastern European AMH	Serbia
SEAMH	SEAMH14	Southeastern European AMH	Serbia
SEAMH	SEAMH15	Southeastern European AMH	Serbia
SEAMH	SEAMH16	Southeastern European AMH	Serbia
SEAMH	SEAMH17	Southeastern European AMH	Serbia
SEAMH	SEAMH18	Southeastern European AMH	Serbia
SEAMH	SEAMH19	Southeastern European AMH	Serbia
SEAMH	SEAMH20	Southeastern European AMH	Serbia
SEAMH	SEAMH21	Southeastern European AMH	Serbia
SEAMH	SEAMH22	Southeastern European AMH	Serbia
SEAMH	SEAMH23	Southeastern European AMH	Serbia
SEAMH	SEAMH24	Southeastern European AMH	Serbia
SEAMH	SEAMH25	Southeastern European AMH	Serbia
SEAMH	SEAMH26	Southeastern European AMH	Serbia
SEAMH	SEAMH27	Southeastern European AMH	Romania
EEAMH	EEAMH1	Eastern European AMH	Ukraine
EEAMH	EEAMH2	Eastern European AMH	Ukraine
EEAMH	EEAMH3	Eastern European AMH	Ukraine
EEAMH	EEAMH4	Eastern European AMH	Ukraine
EEAMH	EEAMH5	Eastern European AMH	Ukraine
EEAMH	EEAMH6	Eastern European AMH	Ukraine
EEAMH	EEAMH7	Eastern European AMH	Ukraine
EEAMH	EEAMH8	Eastern European AMH	Ukraine
EEAMH	EEAMH9	Eastern European AMH	Ukraine

EEAMH	EEAMH10	Eastern European AMH	Ukraine
EEAMH	EEAMH11	Eastern European AMH	Ukraine
EEAMH	EEAMH12	Eastern European AMH	Ukraine
EEAMH	EEAMH13	Eastern European AMH	Ukraine
EEAMH	EEAMH14	Eastern European AMH	Ukraine
EEAMH	EEAMH15	Eastern European AMH	Ukraine
EEAMH	EEAMH16	Eastern European AMH	Ukraine
EEAMH	EEAMH17	Eastern European AMH	Ukraine
EEAMH	EEAMH18	Eastern European AMH	Ukraine
EEAMH	EEAMH19	Eastern European AMH	Ukraine
EEAMH	EEAMH20	Eastern European AMH	Ukraine
EEAMH	EEAMH21	Eastern European AMH	Ukraine
EEAMH	EEAMH22	Eastern European AMH	Ukraine
EEAMH	EEAMH23	Eastern European AMH	Russia
EEAMH	EEAMH24	Eastern European AMH	Russia
EEAMH	EEAMH25	Eastern European AMH	Russia
SE NEANDER		Central European Neanderthals-grouped wi	Croatia
CEAMH	CEAMH1	Central European AMH	Switzerland
CEAMH	CEAMH2	Central European AMH	Germany
CEAMH	CEAMH3	Central European AMH	Czech Republic
CEAMH	CEAMH4	Central European AMH	Germany
CEAMH	CEAMH5	Central European AMH	Czech Republic
CEAMH	CEAMH6	Central European AMH	Czech Republic
CEAMH	CEAMH7	Central European AMH	Czech Republic
CEAMH	CEAMH8	Central European AMH	Czech Republic
CEAMH	CEAMH9	Central European AMH	Czech Republic
CEAMH	CEAMH10	Central European AMH	Germany
CEAMH	CEAMH11	Central European AMH	Germany
CEAMH	CEAMH12	Central European AMH	Czech Republic
CEAMH	CEAMH13	Central European AMH	Switzerland
CEAMH	CEAMH14	Central European AMH	Germany
CEAMH	CEAMH15	Central European AMH	Germany
CEAMH	CEAMH16	Central European AMH	Germany
CEAMH	CEAMH17	Central European AMH	Germany
CEAMH	CEAMH18	Central European AMH	Germany
CEAMH	CEAMH19	Central European AMH	Germany
CEAMH	CEAMH20	Central European AMH	Germany
CEAMH	CEAMH21	Central European AMH	Germany
CEAMH	CEAMH22	Central European AMH	Germany
CEAMH	CEAMH23	Central European AMH	Germany
CEAMH	CEAMH24	Central European AMH	Germany
CEAMH	CEAMH25	Central European AMH	Germany
CEAMH	CEAMH26	Central European AMH	Germany
CEAMH	CEAMH27	Central European AMH	Germany
CEAMH	CEAMH28	Central European AMH	Germany
CEAMH	CEAMH29	Central European AMH	Germany
CEAMH	CEAMH30	Central European AMH	Czech Republic

CEAMH	CEAMH31	Central European AMH	Czech Republic
CEAMH	CEAMH32	Central European AMH	Czech Republic
CEAMH	CEAMH33	Central European AMH	Czech Republic
CEAMH	CEAMH34	Central European AMH	Czech Republic
CEAMH	CEAMH35	Central European AMH	Germany
CEAMH	CEAMH36	Central European AMH	Latvia
CEAMH	CEAMH37	Central European AMH	Latvia
CEAMH	CEAMH38	Central European AMH	Latvia
CEAMH	CEAMH39	Central European AMH	Latvia
CEAMH	CEAMH40	Central European AMH	Latvia
CEAMH	CEAMH41	Central European AMH	Latvia
CEAMH	CEAMH42	Central European AMH	Latvia
CEAMH	CEAMH43	Central European AMH	Latvia
CEAMH	CEAMH44	Central European AMH	Latvia
CEAMH	CEAMH45	Central European AMH	Latvia
CEAMH	CEAMH46	Central European AMH	Latvia
CEAMH	CEAMH47	Central European AMH	Latvia
CEAMH	CEAMH48	Central European AMH	Latvia
CEAMH	CEAMH49	Central European AMH	Latvia
CEAMH	CEAMH50	Central European AMH	Latvia
NEAMH	NEAMH1	Northern European AMH	Norway
NEAMH	NEAMH2	Northern European AMH	Denmark
NEAMH	NEAMH3	Northern European AMH	Denmark
NEAMH	NEAMH4	Northern European AMH	Denmark
NEAMH	NEAMH5	Northern European AMH	Denmark
NEAMH	NEAMH6	Northern European AMH	Denmark
NEAMH	NEAMH7	Northern European AMH	Denmark
NEAMH	NEAMH8	Northern European AMH	Denmark
NEAMH	NEAMH9	Northern European AMH	Denmark
NEAMH	NEAMH10	Northern European AMH	Denmark
NEAMH	NEAMH11	Northern European AMH	Denmark
NEAMH	NEAMH12	Northern European AMH	Denmark
NEAMH	NEAMH13	Northern European AMH	Denmark
NEAMH	NEAMH14	Northern European AMH	Sweden
NEAMH	NEAMH15	Northern European AMH	Sweden
NEAMH	NEAMH16	Northern European AMH	Sweden
NEAMH	NEAMH17	Northern European AMH	Sweden
NEAMH	NEAMH18	Northern European AMH	Sweden
NEAMH	NEAMH19	Northern European AMH	Sweden
NEAMH	NEAMH20	Northern European AMH	Sweden
NEAMH	NEAMH21	Northern European AMH	Sweden
NEAMH	NEAMH22	Northern European AMH	Sweden
NEAMH	NEAMH23	Northern European AMH	Sweden
NEAMH	NEAMH24	Northern European AMH	Denmark
NEAMH	NEAMH25	Northern European AMH	Denmark
NEAMH	NEAMH26	Northern European AMH	Denmark
NEAMH	NEAMH27	Northern European AMH	Denmark

SE NEANDEF	SEN1	Southern European Neanderthals	Spain
SE NEANDEF	SEN2	Southern European Neanderthals	Italy
SE NEANDEF	SEN3	Southern European Neanderthals	Italy
SE NEANDEF	SEN4	Southern European Neanderthals	Italy
SEAMH	SEAMH1	Southern European AMH	Italy
SEAMH	SEAMH2	Southern European AMH	Italy
SEAMH	SEAMH3	Southern European AMH	Italy
SEAMH	SEAMH4	Southern European AMH	Italy
SEAMH	SEAMH5	Southern European AMH	Spain
SEAMH	SEAMH6	Southern European AMH	Italy
SEAMH	SEAMH7	Southern European AMH	Portugal
SEAMH	SEAMH8	Southern European AMH	Portugal
SEAMH	SEAMH9	Southern European AMH	Portugal
SEAMH	SEAMH10	Southern European AMH	Portugal
SEAMH	SEAMH11	Southern European AMH	Portugal
SEAMH	SEAMH12	Southern European AMH	Italy
SEAMH	SEAMH13	Southern European AMH	Italy
SEAMH	SEAMH14	Southern European AMH	Italy
SEAMH	SEAMH15	Southern European AMH	Italy
SEAMH	SEAMH16	Southern European AMH	Italy
SEAMH	SEAMH17	Southern European AMH	Italy
SEAMH	SEAMH18	Southern European AMH	Italy
SEAMH	SEAMH19	Southern European AMH	Italy
SEAMH	SEAMH20	Southern European AMH	Italy
SEAMH	SEAMH21	Southern European AMH	Portugal
SEAMH	SEAMH22	Southern European AMH	Portugal
SEAMH	SEAMH23	Southern European AMH	Portugal
SEAMH	SEAMH24	Southern European AMH	Portugal
SEAMH	SEAMH25	Southern European AMH	Portugal
SEAMH	SEAMH26	Southern European AMH	Portugal
SEAMH	SEAMH27	Southern European AMH	Portugal
SEAMH	SEAMH28	Southern European AMH	Portugal
SEAMH	SEAMH29	Southern European AMH	Portugal
SEAMH	SEAMH30	Southern European AMH	Portugal
SEAMH	SEAMH31	Southern European AMH	Italy
SEAMH	SEAMH32	Southern European AMH	Spain
SEAMH	SEAMH33	Southern European AMH	Italy
SEAMH	SEAMH34	Southern European AMH	Italy
SEAMH	SEAMH35	Southern European AMH	Italy
SEAMH	SEAMH36	Southern European AMH	Italy
SEAMH	SEAMH37	Southern European AMH	Italy
SEAMH	SEAMH38	Southern European AMH	Italy
SEAMH	SEAMH39	Southern European AMH	Italy
WE NEANDE	WEN1	Western European Neanderthals	France
WE NEANDE	WEN2	Western European Neanderthals	France
WE NEANDE	WEN3	Western European Neanderthals	France
WEAMH	WEAMH1	Western European AMH	France

WEAMH	WEAMH2	Western European AMH	France
WEAMH	WEAMH3	Western European AMH	France
WEAMH	WEAMH4	Western European AMH	France
WEAMH	WEAMH5	Western European AMH	France
WEAMH	WEAMH6	Western European AMH	France
WEAMH	WEAMH7	Western European AMH	France
WEAMH	WEAMH8	Western European AMH	France
WEAMH	WEAMH9	Western European AMH	France
WEAMH	WEAMH10	Western European AMH	France
WEAMH	WEAMH11	Western European AMH	France
WEAMH	WEAMH12	Western European AMH	France
WEAMH	WEAMH13	Western European AMH	France
WEAMH	WEAMH14	Western European AMH	France
WEAMH	WEAMH15	Western European AMH	France
WEAMH	WEAMH16	Western European AMH	France
WEAMH	WEAMH17	Western European AMH	France
WEAMH	WEAMH18	Western European AMH	France
WEAMH	WEAMH19	Western European AMH	Luxemborg
WEAMH	WEAMH20	Western European AMH	Belgium
WEAMH	WEAMH21	Western European AMH	Belgium
WEAMH	WEAMH22	Western European AMH	France
WEAMH	WEAMH23	Western European AMH	France
WEAMH	WEAMH24	Western European AMH	France
WEAMH	WEAMH25	Western European AMH	France
WEAMH	WEAMH26	Western European AMH	France
WEAMH	WEAMH27	Western European AMH	France
WEAMH	WEAMH28	Western European AMH	France
WEAMH	WEAMH29	Western European AMH	France
WEAMH	WEAMH30	Western European AMH	France
WEAMH	WEAMH31	Western European AMH	France
WEAMH	WEAMH32	Western European AMH	France
WEAMH	WEAMH33	Western European AMH	France
WEAMH	WEAMH34	Western European AMH	France
WEAMH	WEAMH35	Western European AMH	France
WEAMH	WEAMH36	Western European AMH	France
WEAMH	WEAMH37	Western European AMH	France
WEAMH	WEAMH38	Western European AMH	France
WEAMH	WEAMH39	Western European AMH	France
WEAMH	WEAMH40	Western European AMH	France
WEAMH	WEAMH41	Western European AMH	France
WEAMH	WEAMH42	Western European AMH	France
WEAMH	WEAMH43	Western European AMH	U.K.
WEAMH	WEAMH44	Western European AMH	U.K.
NE/NAAMH	MENAAMH1	Middle Eastern-North Africa AMNH	
NE/NAAMH	MENAAMH2	Middle Eastern-North Africa AMNH	
NE/NAAMH	MENAAMH3	Middle Eastern-North Africa AMNH	

Specimen	Latitude	Longitude	Date	CZ	
Amud 1	32.87	35.51	45000	4	cool temperate moist
Shanidar 1	36.8	44.24	55000	4	cool temperate moist
Shanidar 4 (recon)	36.8	44.24	55000	4	cool temperate moist
Shanidar 5	36.8	44.24	55000	4	cool temperate moist
Tabun 1	32.74	35.04	120000	3	warm temperate moist
Qafzeh 6	32.41	35.19	100000	3	warm temperate moist
Qafzeh 9	32.41	35.19	100000	3	warm temperate moist
Skhul 4	32.41	35.19	100000	3	warm temperate moist
Skhul 5	32.41	35.19	100000	3	warm temperate moist
Skhul 9	32.41	35.19	100000	3	warm temperate moist
Franchthi 1	37.42	23.12	.	4	cool temperate moist
Franchthi 2	37.42	23.12	.	4	cool temperate moist
Muierii 1	45.18	23.77	29930	4	cool temperate moist
Padina 26	44.63	21.98	.	4	cool temperate moist
Theopetra 2	39.68	21.68	8070	4	cool temperate moist
Vlasac 27	44.31	22.01	8870	4	cool temperate moist
Vlasac 29	44.31	22.01	8870	4	cool temperate moist
Vlasac 36	44.31	22.01	8870	4	cool temperate moist
Vlasac 38	44.31	22.01	8870	4	cool temperate moist
Vlasac 40	44.31	22.01	8870	4	cool temperate moist
Vlasac 43	44.31	22.01	8870	4	cool temperate moist
Vlasac 45	44.31	22.01	8870	4	cool temperate moist
Vlasac 46	44.31	22.01	8870	4	cool temperate moist
Vlasac 48	44.31	22.01	8870	4	cool temperate moist
Vlasac 4A	44.31	22.01	8870	4	cool temperate moist
Vlasac 55	44.31	22.01	8870	4	cool temperate moist
Vlasac 56	44.31	22.01	8870	4	cool temperate moist
Vlasac 6	44.31	22.01	8870	4	cool temperate moist
Vlasac 60	44.31	22.01	8870	4	cool temperate moist
Vlasac 67	44.31	22.01	8870	4	cool temperate moist
Vlasac 69	44.31	22.01	8870	4	cool temperate moist
Vlasac 69A	44.31	22.01	8870	4	cool temperate moist
Vlasac 77	44.31	22.01	8870	4	cool temperate moist
Vlasac 79	44.31	22.01	8870	4	cool temperate moist
Vlasac 82	44.31	22.01	8870	4	cool temperate moist
Vlasac 83	44.31	22.01	8200	4	cool temperate moist
Oase 2	45.01667	21.83333	34950	4	cool temperate moist
Vasilevka III-10	48	35.06	10040	5	cold and mesic
Vasilevka III-14	48	35.06	10040	5	cold and mesic
Vasilevka III-18	48	35.06	10040	5	cold and mesic
Vasilevka III-19	48	35.06	10040	5	cold and mesic
Vasilevka III-1953	48	35.06	10040	5	cold and mesic
Vasilevka III-22	48	35.06	10040	5	cold and mesic
Vasilevka III-23	48	35.06	10040	5	cold and mesic
Vasilevka III-24	48	35.06	10040	5	cold and mesic
Vasilevka III-25	48	35.06	10040	5	cold and mesic

Vasilevka III-26	48	35.06	10040	5	cold and mesic
Vasilevka III-28	48	35.06	10040	5	cold and mesic
Vasilevka III-31	48	35.06	10040	5	cold and mesic
Vasilevka III-33	48	35.06	10040	5	cold and mesic
Vasilevka III-34	48	35.06	10040	5	cold and mesic
Vasilevka III-36	48	35.06	10040	5	cold and mesic
Vasilevka III-38	48	35.06	10040	5	cold and mesic
Vasilevka III-8	48	35.06	10040	5	cold and mesic
Vasilevka IV-1	48	35.06	10000	5	cold and mesic
Vasilevka1-13	48	35.06	10000	5	cold and mesic
Vasilevka1-17	48	35.06	10000	5	cold and mesic
Vasilevka1-20	48	35.06	10000	5	cold and mesic
Murzak Koba	44.64	35.52	7390	5	cold and mesic
Kostenki2	51.39	39.05	27915	5	cold and mesic
Sunghir 1	56.13	40.42	27050	5	cold and mesic
Sunghir 5	56.13	40.42	26525	5	cold and mesic
Krapi 3	46.16	15.87	130000	1	cool temperate mesic
Birsmatten 1	47.44	7.55	6290	6	cool temperate and dry
Bottendorf 1	41.39	9.16	5950	6	cool temperate and dry
Brno 3	49.19	16.61	15215	6	cool temperate and dry
Dobritz 2	50.69	11.65	8400	6	cool temperate and dry
Dolni Vestonice 13	48.88	16.64	26640	6	cool temperate and dry
Dolni Vestonice 14	48.88	16.64	26640	6	cool temperate and dry
Dolni Vestonice 15	48.88	16.64	26640	6	cool temperate and dry
Dolni Vestonice 16	48.88	16.64	26640	6	cool temperate and dry
Dolni Vestonice 3	48.88	16.64	25950	6	cool temperate and dry
Durrenberg 1	51.3	12.07	7745	6	cool temperate and dry
Groß Fredenwalde K2/craniu	53.12	13.8	7390	6	cool temperate and dry
Mladec 1	49.71	17.01	31190	6	cool temperate and dry
Le Bichon 1	47.1	6.87	11685	6	cool temperate and dry
Oberkassel 1	50.71	7.16	11570	6	cool temperate and dry
Oberkassel 2	50.71	7.16	12180	6	cool temperate and dry
Ofnet 11	48.82	10.45	7515	6	cool temperate and dry
Ofnet 13	48.82	10.45	7515	6	cool temperate and dry
Ofnet 14	48.82	10.45	7515	6	cool temperate and dry
Ofnet 15	48.82	10.45	7515	6	cool temperate and dry
Ofnet 18	48.82	10.45	7515	6	cool temperate and dry
Ofnet 2	48.82	10.45	7515	6	cool temperate and dry
Ofnet 21	48.82	10.45	7515	6	cool temperate and dry
Ofnet 24	48.82	10.45	7515	6	cool temperate and dry
Ofnet 25	48.82	10.45	7515	6	cool temperate and dry
Ofnet 29	48.82	10.45	7515	6	cool temperate and dry
Ofnet 3	48.82	10.45	7520	6	cool temperate and dry
Ofnet 32	48.82	10.45	7560	6	cool temperate and dry
Ofnet 4	48.82	10.45	7515	6	cool temperate and dry
Ofnet 8	48.82	10.45	7360	6	cool temperate and dry
Predmosti 10	49.47	17.44	26595	6	cool temperate and dry

Predmosti 3	49.47	17.44	26595	6	cool temperate and dry
Predmosti 4	49.47	17.44	26595	6	cool temperate and dry
Predmosti 7	49.47	17.44	26595	6	cool temperate and dry
Predmosti 9	49.47	17.44	26595	6	cool temperate and dry
Unseburg 1	55.58	11.48	7670	6	cool temperate and dry
Zvejnieki121	57.82	25.17	11871	6	cool temperate and dry
Zvejnieki122	57.82	25.17	11871	6	cool temperate and dry
Zvejnieki154	57.82	25.17	11871	6	cool temperate and dry
Zvejnieki16	57.82	25.17	11871	6	cool temperate and dry
Zvejnieki165	57.82	25.17	11871	6	cool temperate and dry
Zvejnieki17	57.82	25.17	11871	6	cool temperate and dry
Zvejnieki2	57.82	25.17	11871	6	cool temperate and dry
Zvejnieki35	57.82	25.17	11871	6	cool temperate and dry
Zvejnieki37	57.82	25.17	11871	6	cool temperate and dry
Zvejnieki57	57.82	25.17	11871	6	cool temperate and dry
Zvejnieki71	57.82	25.17	11871	6	cool temperate and dry
Zvejnieki74	57.82	25.17	11871	6	cool temperate and dry
Zvejnieki76	57.82	25.17	11871	6	cool temperate and dry
Zvejnieki9	57.82	25.17	11871	6	cool temperate and dry
ZvejniekiNN1	57.82	25.17	11871	6	cool temperate and dry
Bleivik 1	54.6	11	7950	5	cold and mesic
Bøgebakken 10	55.77	12.58	6310	5	cold and mesic
Bøgebakken 22	55.77	12.58	6310	5	cold and mesic
Bøgebakken 2	55.77	12.58	6310	5	cold and mesic
Bonifacio 2	55.77	12.58	8250	5	cold and mesic
Fannerup 1	56.4	10.71	.	5	cold and mesic
Koed 1	56.38	10.57	6200	5	cold and mesic
Koelbjerg 1	55.4	10.13	9285	5	cold and mesic
Korsør Glasværk AS 74-45	55.35	11.16	6180	5	cold and mesic
Korsør Nor 1	55.35	11.16	6263	5	cold and mesic
Korsør Nor 2	55.35	11.16	6263	5	cold and mesic
Melby 1	55.93	11.98	6280	5	cold and mesic
Sejerø 1	55.91	11.09	5360	5	cold and mesic
Skateholm I - 14B	55.38	13.48	6255	5	cold and mesic
Skateholm I - 22	55.38	13.48	6255	5	cold and mesic
Skateholm I - 33	55.38	13.48	6255	5	cold and mesic
Skateholm I - 37	55.38	13.48	6270	5	cold and mesic
Skateholm I - 4	55.38	13.48	6255	5	cold and mesic
Skateholm I - 6	55.38	13.48	6255	5	cold and mesic
Skateholm I - 7	55.38	13.48	6255	5	cold and mesic
Skateholm II - IX	55.38	13.48	6170	5	cold and mesic
Skateholm II - XXII	55.38	13.48	6170	5	cold and mesic
Store Bjers 1	57.8	18.53	7970	5	cold and mesic
Strøby Egede A	56	13.62	6500	5	cold and mesic
Strøby Egede D	55.41	12.24	6500	5	cold and mesic
Vedbæk-Boldbaner 1	55.85	12.56	7115	5	cold and mesic
Vænge Sø 2	56.13	10.52	5500	5	cold and mesic

Gilbraltr 1 (Forbes Quarry)	36.14	-5.34	58000	2	warm temperate mesic
Guattari 1 (Il Circeo 2)	41.23	13.04	55000	2	warm temperate mesic
Saccopastore 1	41.93	12.52	120000	2	warm temperate mesic
Saccopastore 2	41.93	12.52	120000	2	warm temperate mesic
Arene Candide 1	44.17	8.34	10450	2	warm temperate mesic
Arene Candide 19	44.17	8.34	10450	2	warm temperate mesic
Arene Candide 4	44.17	8.34	10450	2	warm temperate mesic
Arene Candide 5	44.17	8.34	10450	2	warm temperate mesic
Brana 1	42.95	-5.37	6980	2	warm temperate mesic
Maritza 2	41.9	13.65	10600	2	warm temperate mesic
Moita do Sebastiao 1 (1952/	39.11	-8.68	7120	2	warm temperate mesic
Moita do Sebastiao 12 (Crar	39.11	-8.68	7120	2	warm temperate mesic
Moita do Sebastiao 19 (Crar	39.11	-8.68	7120	2	warm temperate mesic
Moita do Sebastiao 20 (Crar	39.11	-8.68	7120	2	warm temperate mesic
Moita do Sebastiao 3 (1952/	39.11	-8.68	7120	2	warm temperate mesic
Ortucchio 1	41.94	13.63	12619	2	warm temperate mesic
Paglicci 25	41.65	15.61	23255	2	warm temperate mesic
Romito 4	39.87	15.91	10862	2	warm temperate mesic
Romito 5	39.87	15.91	11340	2	warm temperate mesic
Romito 6	39.87	15.91	11340	2	warm temperate mesic
San Teodoro 1	38.05	14.58	12580	2	warm temperate mesic
San Teodoro 2	38.05	14.58	12580	2	warm temperate mesic
San Teodoro 3	38.05	14.58	12580	2	warm temperate mesic
San Teodoro 6	38.05	14.58	12580	2	warm temperate mesic
Moita do Sebastiao 9 (Ossa	39.11	-8.68	7120	2	warm temperate mesic
Moita do Sebastiao VII (Crar	39.11	-8.68	7120	2	warm temperate mesic
Moita do Sebastiao XLI (Cra	39.11	-8.68	7120	2	warm temperate mesic
Moita do Sebastiao XVI (Cra	39.11	-8.68	7120	2	warm temperate mesic
Moita do Sebastiao XVII (Cr	39.11	-8.68	7120	2	warm temperate mesic
Moita do Sebastiao XVIII (Cr	39.11	-8.68	7120	2	warm temperate mesic
Moita do Sebastiao XXII (Cr	39.11	-8.68	7120	2	warm temperate mesic
Moita do Sebastiao XXXII (C	39.11	-8.68	7120	2	warm temperate mesic
Moita do Sebastiao XXXIX (	39.11	-8.68	7120	2	warm temperate mesic
Moita do Sebastiao XXXV (C	39.11	-8.68	7120	2	warm temperate mesic
Molara 1	38.08	13.31	8600	2	warm temperate mesic
Nerja 5	36.77	-3.84	8260	2	warm temperate mesic
Uzzo 1A	38.11	12.78	9270	2	warm temperate mesic
Uzzo 1B	38.11	12.78	9270	2	warm temperate mesic
Uzzo 4A	38.11	12.78	9270	2	warm temperate mesic
Uzzo 4B	38.11	12.78	9270	2	warm temperate mesic
Uzzo 5	38.11	12.78	9270	2	warm temperate mesic
Uzzo 7	38.11	12.78	9270	2	warm temperate mesic
Vatte di Zamba 1	46.16	11.08	7980	2	warm temperate mesic
La Chapelle-aux-Saints 1	44.98	1.72	50000	4	cool temperate moist
La Ferrassie 1	44.95	0.94	70000	4	cool temperate moist
Saint-Cesaire 1	45.75	-0.5	36000	4	cool temperate moist
Abri Pataud 1	44.94	1.01	20535	4	cool temperate moist

Baume de Montclus 1	44.27	4.42	6665	4	cool temperate moist
Bruniquel 24	44.05	1.67	15290	4	cool temperate moist
Cap Blanc 1	44.93	1.08	.	4	cool temperate moist
Chancelade 1	45.22	0.68	.	4	cool temperate moist
Combe Capelle 1	44.75	0.85	8561	4	cool temperate moist
Cro Magnon 2	44.94	1.01	27680	4	cool temperate moist
Cro Magnon 3	44.94	1.01	27680	4	cool temperate moist
Culoz 1	45.84	5.58	.	4	cool temperate moist
Culoz 2	45.84	5.58	.	4	cool temperate moist
Gramat 1	44.75	1.68	.	4	cool temperate moist
Hoedic 1 (B)	47.35	-2.86	5750	4	cool temperate moist
Hoedic 6	47.35	-2.86	6280	4	cool temperate moist
Hoedic 8 (H)	47.35	-2.86	6080	4	cool temperate moist
Hoedic 9 (K)	47.35	-2.86	5755	4	cool temperate moist
Laugerie-Basse 4	44.95	1	15660	4	cool temperate moist
Le Peyrat 5	45.17	1.15	11430	4	cool temperate moist
Le Placard 5 (1881)	45.69	0.42	.	4	cool temperate moist
Loschbour 1	49.76	6.28	7205	4	cool temperate moist
Malonne 1	50.43	4.8	.	4	cool temperate moist
Malonne 140	50.43	4.8	9270	4	cool temperate moist
Montgaudier 4	45.67	0.49	11930	4	cool temperate moist
Rochereil 0	45.3	0.54	.	4	cool temperate moist
Rochereil 1	45.3	0.54	.	4	cool temperate moist
Rond-du-Barry 8	45.07	3.87	17100	4	cool temperate moist
St. Germain-la-Riviere 1	44.95	-0.33	15780	4	cool temperate moist
Teviec 1 (D1)	47.56	-3.16	6510	4	cool temperate moist
Teviec 10 (K4)	47.56	-3.16	6510	4	cool temperate moist
Teviec 11 (E1)	47.56	-3.16	6510	4	cool temperate moist
Teviec 13 (M)	47.56	-3.16	6740	4	cool temperate moist
Teviec 14 (H1)	47.56	-3.16	6515	4	cool temperate moist
Teviec 15 (H3)	47.56	-3.16	6530	4	cool temperate moist
Teviec 16 (K6)	47.56	-3.16	6500	4	cool temperate moist
Teviec 18	47.56	-3.16	6510	4	cool temperate moist
Teviec 2 (B)	47.56	-3.16	6322	4	cool temperate moist
Teviec 3	47.56	-3.16	6510	4	cool temperate moist
Teviec 4	47.56	-3.16	6510	4	cool temperate moist
Teviec 6	47.56	-3.16	6510	4	cool temperate moist
Teviec 7 (K2)	47.56	-3.16	6510	4	cool temperate moist
Teviec 8 (K1)	47.56	-3.16	6440	4	cool temperate moist
Teviec 9 (K3)	47.56	-3.16	6510	4	cool temperate moist
Veyrier 2	46.15	6.18	10630	4	cool temperate moist
Aveline's Hole "A"	51.33	-2.75	9085	4	cool temperate moist
Aveline's Hole "B"	51.33	-2.75	9085	4	cool temperate moist
Wadi Kubbaniya	21.1479	78.9747	20000	7	warm temperate xeric
Ohalo II	32.72209	35.57214	19000	7	warm temperate xeric
Nahal ein Gev 1	32.7687	35.6377	12000	7	warm temperate xeric

	UFH (N-P)	FMB	NLH	NLB	OBB	OBH	GM	NPs
Frumkin et al. 2011	89	114	64	.	44	37	63.80076	1.394968
Frumkin et al. 2011	86	115	62	31	47	36	56.39297	1.525013
Frumkin et al. 2011	93	118	.	32	.	37	60.03841	1.549008
Frumkin et al. 2011	94	116	69	38.5	47	37	60.7714	1.54678
Nichols 2017	79	107	58.2	34	.	35	56.68866	1.393577
Nichols 2017	75	110	52	32	47	35	53.16537	1.410693
Nichols 2017	72	103	54	30	42	29	49.45634	1.455829
Nichols 2017	79	106	55	30	44	34	52.38715	1.508003
Nichols 2017	73	99	53	28	44	30	49.18443	1.48421
Nichols 2017	74	96	55	30	44	37	51.69401	1.4315
Metzger et al 2012	69	100	50	24	.	31	48.07047	1.435393
Metzger et al 2012	69	100	51	26	.	31	49.04005	1.407013
Metzger et al 2012	70	.	48	25	43.5	31.5	40.94641	1.709552
Metzger et al 2012	65	.	54	24	40	23	37.83217	1.718115
Metzger et al 2012	70	87	53	21	.	34	47.04552	1.487921
Metzger et al 2012	63	103	48	27	44	33	47.98714	1.312852
Metzger et al 2012	66	95	51	26	42	32	47.28264	1.395861
Metzger et al 2012	.	93	47	22	41	32	41.70494	.
Metzger et al 2012	68	96	48	22	41	33	45.88002	1.482126
Metzger et al 2012	75	95	64	26	44	36	51.55636	1.454719
Metzger et al 2022	.	104	.	26	42	34	44.32857	.
Metzger et al 2023	79	.	55	28	44	33	44.60904	1.770941
Metzger et al 2024	.	103	.	26	42	34	44.22162	.
Metzger et al 2025	75	98	54	22	43	36	48.80666	1.536675
Metzger et al 2026	71	102	.	.	42	35	57.12076	1.242981
Metzger et al 2027	72	96	53	25	43	36	49.19622	1.463527
Metzger et al 2028	62	90	49	.	.	34	55.21757	1.122831
Metzger et al 2029	80	101	57	.	46	37	60.0965	1.331192
Metzger et al 2030	78	103	55	24	.	.	57.06587	1.366842
Metzger et al 2031	69	107	52	24	.	.	55.09496	1.252383
Metzger et al 2032	68	97	51	25	39	32	46.79156	1.453253
Metzger et al 2033	73	101	55	30	.	.	59.05845	1.236064
Metzger et al 2034	64	93	48	23	45	31	45.74754	1.398982
Metzger et al 2035	67	107	48	24	43	30	46.90835	1.428317
Metzger et al 2036	67	97	51	25	41	29	46.30094	1.447055
Metzger et al 2037	66	97	48	25	.	29	46.72819	1.412424
Metzger et al 2038	69	107.5	47.8	25	31.5	41.7	47.60786	1.44934
Metzger et al 2039	62	90	47	25	40	33	45.31142	1.368308
Metzger et al 2040	69	93	52	25	39	35	47.43163	1.454726
Metzger et al 2041	71	98	53	23	45	27	46.64919	1.521999
Metzger et al 2042	67	90	54	20	41	34	45.67382	1.466924
Metzger et al 2043	71	101	51	23	41	32	47.18478	1.504723
Metzger et al 2044	63	81	50	23	40	32	44.25431	1.42359
Metzger et al 2045	70	89	54	25	44	32	47.74238	1.466203
Metzger et al 2046	68	94	47	24	47	33	47.28894	1.437968
Metzger et al 2047	74	103	55	32	46	34	52.51738	1.409057

Metzger et al 2048	70	95	53	24	41	30	46.72353	1.498174
Metzger et al 2049	68	93	52	24	40	35	47.19425	1.440854
Metzger et al 2050	77	94	58	.	46	34	58.00383	1.327499
Metzger et al 2051	70	90	55	20	42	34	46.33511	1.510733
Metzger et al 2052	69	102	53	27	.	32	50.30924	1.371517
Metzger et al 2053	70	94	52	21	39	28	44.57711	1.570313
Metzger et al 2054	72	97	52	24	46	31	48.12895	1.495981
Metzger et al 2055	64	98	52	23	42	31	46.23363	1.384274
Metzger et al 2056	69	95	51	22	41	34	46.60917	1.480395
Metzger et al 2057	74	100	56	27.7	43	33	50.34778	1.469777
Metzger et al 2058	80	93	56	26.5	43	35	50.51541	1.583675
Metzger et al 2059	78	100	55	25	44	35	50.46454	1.54564
Metzger et al 2060	76	98	52	.	47	27	54.73951	1.388394
Metzger et al 2061	60	93	43	27	43	27	44.26391	1.355506
Metzger et al 2062	78	102	56	27	46	34	51.57199	1.512449
Metzger et al 2063	63	104	46	25	45	30	46.54799	1.353442
Nichols 2017	111	.	.	27.5	44	37	47.21473	2.350961
Metzger et al 2012	66	89	50	22	40	30	44.48886	1.483517
Metzger et al 2013	66	89	50	22	40	30	44.48886	1.483517
Metzger et al 2014	65	92	49	26	40	33	46.45945	1.399069
Metzger et al 2015	.	91	56	26	41	26	42.65717	.
Metzger et al 2016	69	100	54	30	42	31	49.41163	1.396432
Metzger et al 2017	68	105	56	27	44	28	48.67547	1.397008
Metzger et al 2018	63	99	50	22	42	31	45.55086	1.383069
Metzger et al 2019	70	97	59	28	.	32	51.40507	1.361733
Metzger et al 2020	63	92	52	23	41	31	45.44654	1.386244
Metzger et al 2021	63	89	47	24	41	30	44.5134	1.415304
Metzger et al 2022	.	95	45	.	40	30	47.59149	.
Metzger et al 2023	70	104	50	26	40	32	47.92338	1.460665
Metzger et al 2024	68	100	48	26	43	30	47.12147	1.443079
Metzger et al 2025	72	98	47	30	51	25	48.29268	1.490909
Metzger et al 2026	64	92	40	31	46	25	45.08318	1.419598
Metzger et al 2027	64	100	51	28	.	.	54.98282	1.164
Metzger et al 2028	64	92	48	23	38	33	44.86136	1.426618
Metzger et al 2029	58	96	46	24	42	29	44.23024	1.31132
Metzger et al 2030	72	93	54	24	38	27	45.52619	1.581507
Metzger et al 2031	76	96	58	22	43	31	48.11653	1.579499
Metzger et al 2032	67	.	51	21	34	30	37.40183	1.791356
Metzger et al 2033	75	98	42	26	46	31	47.47208	1.579876
Metzger et al 2034	55	103	45	25	41	32	45.05203	1.220811
Metzger et al 2035	65	93	49	24	40	31	45.45038	1.430131
Metzger et al 2036	67	90	52	26	37	31	45.89967	1.459705
Metzger et al 2037	61	91	41	26	35	.	46.05102	1.324618
Metzger et al 2038	56	92	44	24	42	28	43.0865	1.299711
Metzger et al 2039	72	93	58	.	39	30	53.88723	1.336124
Metzger et al 2040	58	92	41	25	39	30	43.08779	1.346089
Metzger et al 2041	68	103	51	28	41	27	47.21033	1.440363

Metzger et al 2042	77	104	59	25	42	29	49.31669	1.561338
Metzger et al 2043	63	98	48	27	38	27	44.91449	1.402665
Metzger et al 2044	.	101	.	29	39	28	42.28979	.
Metzger et al 2045	68	105	54	25	39	26	46.23934	1.470609
Metzger et al 2046	70	93	47	24	41	30	45.63512	1.533906
Metzger et al 2047	67	89	48	24	42	34	46.26731	1.448107
Metzger et al 2048	66	96	47	25	44.5	31	46.62252	1.415625
Metzger et al 2049	75	94	54	25	42	28	47.2957	1.585768
Metzger et al 2050	73	92	53	25	43.8	34	48.64594	1.500639
Metzger et al 2051	63	102	50	25	45	34	48.0386	1.311445
Metzger et al 2052	66	92	49	23	42	32	45.77298	1.441899
Metzger et al 2053	80	94	60	26	45	32	50.65444	1.579329
Metzger et al 2054	.	102	52	25	47	33	45.98657	.
Metzger et al 2055	68	92	48	24.5	46.5	35	47.83042	1.421689
Metzger et al 2056	66	89	48	23	42	28	44.36615	1.487621
Metzger et al 2057	71	100	55	25	38	29	46.98477	1.511128
Metzger et al 2058	71	96	51	22.5	42	34	47.27901	1.501723
Metzger et al 2059	61	93	47	23	41	30	44.28506	1.37744
Metzger et al 2060	71	94	50	22	44	33	46.91275	1.513448
Metzger et al 2061	71	104	52	26	41	32	48.55124	1.462372
Metzger et al 2062	72	92	50	23	.	31	47.27548	1.522988
Metzger et al 2063	74	111	55	27	49	31	51.44065	1.438551
Metzger et al 2064	69	94	50	22	39	32	45.52648	1.515602
Metzger et al 2065	75	96	54	23	42	34	48.34635	1.551306
Metzger et al 2066	68	100	47	25	41	28	45.75249	1.486258
Metzger et al 2067	.	96	52	25	.	28	43.23577	.
Metzger et al 2068	65	94	44	22	.	31	44.94234	1.446298
Metzger et al 2069	70	101	50	20	42	32	46.02245	1.520997
Metzger et al 2070	65	103	47	25	.	28	46.62183	1.394197
Metzger et al 2071	78	106	58	24	44	32	50.30453	1.550556
Metzger et al 2072	.	102	50	25	.	29	43.85075	.
Metzger et al 2073	74	101	53	23	45	31	48.30831	1.531828
Metzger et al 2074	73	97	53	29	.	32	51.09562	1.428694
Metzger et al 2075	72	105	52	22	.	.	54.22967	1.327687
Metzger et al 2076	82	106	57	28	.	36	54.915	1.493217
Metzger et al 2077	70	96	51	22	.	.	52.40112	1.335849
Metzger et al 2078	65	96	52	24	.	37	49.19491	1.321275
Metzger et al 2079	71	102	53	26	42	33	48.99426	1.449149
Metzger et al 2080	72	99	50	22	41	34	47.109	1.52837
Metzger et al 2081	77	102	57	24	.	.	57.25247	1.34492
Metzger et al 2082	74	99	50	24	43	32	47.91201	1.544498
Metzger et al 2083	62	102	.	.	42	30	53.1301	1.166947
Metzger et al 2084	71	103	52	22	45	34	48.36548	1.467989
Metzger et al 2085	.	108	56	23	.	37	47.63052	.
Metzger et al 2086	79	106	53	26	.	34	52.32777	1.509715
Metzger et al 2087	72	102	50	24	44	31	47.86169	1.504335
Metzger et al 2088	66	101	50	26	41	34	47.90107	1.37784

Nichols 2017	75	108	58	38	43	38	55.48277	1.351771
Nichols 2018	87	111	62	37	44	37	57.47977	1.513576
Nichols 2017	78	118	60	31.2	43	37	54.91093	1.420482
Nichols 2017	89	123	63	33.6	46	34	57.52659	1.547111
Metzger et al 2012	67	87	50	24	45	32	46.47181	1.441734
Metzger et al 2013	67	107	46	21	47	32	46.73195	1.433709
Metzger et al 2014	67	96	51	21	45	35	47.05105	1.423985
Metzger et al 2015	69	100	50	24	49	37	49.66739	1.389241
Metzger et al 2016	72	92	50	23	.	31	47.27548	1.522988
Metzger et al 2017	64	99	48	27	46	30	47.39326	1.350403
Metzger et al 2018	72	93	50	22	.	29	46.33629	1.553858
Metzger et al 2019	63	95	45	22	40	29	43.60399	1.444822
Metzger et al 2020	64	97	46	24	39	30	44.73881	1.430525
Metzger et al 2021	67	87	48	27	39	28	44.95075	1.49052
Metzger et al 2022	73	94	50	26	37	31	46.5936	1.566739
Metzger et al 2023	61	90	46	24	43	31	44.7949	1.361762
Metzger et al 2024	68	101	50	24	40	29	46.0693	1.476037
Metzger et al 2025	68	94	51	23	41	28	45.27024	1.502091
Metzger et al 2026	.	84	43	24	39	28	39.37643	.
Metzger et al 2027	65	92	49	27	42	28	45.86113	1.417322
Metzger et al 2028	68	97	47	24	42	30	45.9191	1.480865
Metzger et al 2029	66	92	48	24.5	44	28	45.43512	1.452621
Metzger et al 2030	70	92	53	25	41	30	46.79159	1.495995
Metzger et al 2031	58	93	46	20	40	30	42.57422	1.362327
Metzger et al 2032	72	.	50	23	41	37	41.66805	1.727943
Metzger et al 2033	70	90	48	23	40	30	45.03828	1.554233
Metzger et al 2034	72	100	53	25	.	.	55.57598	1.295524
Metzger et al 2035	64	87	45	26	39	28	43.85488	1.459359
Metzger et al 2036	62	89	43	22	42	28	42.79043	1.448922
Metzger et al 2037	62	87	43	23	40	29	42.8477	1.446985
Metzger et al 2038	64	.	43	26	.	28	37.62227	1.70112
Metzger et al 2039	75	105	49	28	46	28	49.0441	1.529236
Metzger et al 2040	72	98	52	23	.	.	53.89797	1.335857
Metzger et al 2041	66	93	.	21	38	26	41.7831	1.579586
Metzger et al 2042	75	98	52	22	45	32	47.91954	1.565124
Metzger et al 2043	68	87	49	25	.	30	46.50044	1.462352
Metzger et al 2044	70	99	52	22	42	33	47.15058	1.484605
Metzger et al 2045	70	98	53	21	43	30	46.29863	1.511924
Metzger et al 2046	64	95	52	19	43	28	43.97588	1.455343
Metzger et al 2047	68	96	52	22	42	34	46.91615	1.449394
Metzger et al 2048	62	92	46	22	43	27	43.42089	1.427884
Metzger et al 2049	73	95	55	.	39	31	54.04644	1.35069
Metzger et al 2050	62	92	46	24	40	28	43.79197	1.415785
Pederazi et al. 2011	82	114	61	34	46	38	56.8868	1.441459
Pederazi et al. 2011	91	112	60	34.3	45	36	56.91193	1.598962
Pederazi et al. 2011	83	114	60	27.5	42	36	53.55949	1.549679
Metzger et al 2012	66	99	49	24	42	32	46.66568	1.414316

Metzger et al 2013	75	96	54	23	42	34	48.34635	1.551306
Metzger et al 2014	67	94	46	21	36	29	43.03416	1.556903
Metzger et al 2015	75	99	56	23	.	.	55.61002	1.348678
Metzger et al 2016	77	101	58	28	40	32	50.28456	1.531285
Metzger et al 2017	79	91	56	30	46	33	51.35013	1.538457
Metzger et al 2018	66	101	52	24	47	27	46.83713	1.409139
Metzger et al 2019	67	102	54	27	43	31	48.6643	1.376779
Metzger et al 2020	54	112	42	22	44	27	43.35275	1.245596
Metzger et al 2021	71	92	50	28	44	33	48.66202	1.459043
Metzger et al 2022	75	101	55	26	45	32	49.98582	1.500425
Metzger et al 2023	71	104	52	26	41	31	48.29502	1.470131
Metzger et al 2024	73	104	53	28	42	29	48.92974	1.491935
Metzger et al 2025	64	100	49	23	36	30	44.52336	1.437448
Metzger et al 2026	70	108	50	28	44	34	50.11067	1.396908
Metzger et al 2027	70	98	.	26	.	.	56.29016	1.243557
Metzger et al 2028	72	96	55	25.5	39	31	47.66016	1.510696
Metzger et al 2029	58	90	43	22	36	26	40.81373	1.421091
Metzger et al 2030	65	100	43	24	39	30	44.57895	1.458087
Metzger et al 2031	64	92	44	23	40	28	43.39047	1.474978
Metzger et al 2032	64	92	44	23	.	.	49.40684	1.295367
Metzger et al 2033	66	98	51	27	41	31	47.38508	1.392844
Metzger et al 2034	72	97	52	24	42	29	46.88073	1.535812
Metzger et al 2035	72	97	52	24	42	30	47.14637	1.527159
Metzger et al 2036	71	99	51	23	48	31	48.02482	1.478402
Metzger et al 2037	67	98	53	24	38	31	46.29018	1.447391
Metzger et al 2038	63	98	47	23	39	28	44.03208	1.430775
Metzger et al 2039	66	96	48	23	41	30	45.26698	1.458016
Metzger et al 2040	74	102	54	28	42	30	49.31273	1.500627
Metzger et al 2041	75	104	56	28	45	35	51.77501	1.448575
Metzger et al 2042	68	98	46	24	38	30	45.0749	1.5086
Metzger et al 2043	68	97	47	21	39	31	44.60026	1.524655
Metzger et al 2044	73	98	56	25	44	32	49.15261	1.48517
Metzger et al 2045	67	93	53	26	38	32	46.75091	1.433127
Metzger et al 2046	64	91	46	23	39	28	43.44985	1.472963
Metzger et al 2047	62	103	47	22	42	27	44.23103	1.401731
Metzger et al 2048	62	95	47	25	.	29	45.76255	1.35482
Metzger et al 2049	63	98	49	25	38	30	45.28294	1.391252
Metzger et al 2050	65	100	52	26	.	30	48.32838	1.344965
Metzger et al 2051	74	94	52	24	43	31	47.55915	1.555957
Metzger et al 2052	70	102	50	24	43	31	47.45532	1.475072
Metzger et al 2053	62	100	45	23	39	27	43.48039	1.42593
Metzger et al 2054	69	94	50	22	39	32	45.52648	1.515602
Metzger et al 2055	.	96	.	25	39	31	41.27237	.
Metzger et al 2056	68	.	44	27	46	30	40.68562	1.671352
Metzger et al 2057	66	107	.	25	40	28	45.6265	1.446528
Metzger et al 2058	60	100	46	27	39	27	44.57762	1.345967

FMBs	NLHs	NLBs	OBBs	OBHs
1.786813	1.003123	.	0.689647	0.57993
2.039261	1.099428	0.549714	0.833437	0.638377
1.965409	.	0.532992	.	0.616272
1.908793	1.135403	0.633522	0.77339	0.608839
1.887503	1.02666	0.599767	.	0.617407
2.069016	0.97808	0.601896	0.884034	0.658323
2.082645	1.091872	0.606596	0.849234	0.586376
2.023397	1.049876	0.57266	0.839901	0.649014
2.012832	1.077577	0.569286	0.894592	0.609949
1.857082	1.063953	0.580338	0.851162	0.71575
2.080279	1.04014	0.499267	.	0.644887
2.03915	1.039966	0.530179	.	0.632136
.	1.172264	0.610554	1.062364	0.769298
.	1.427357	0.634381	1.057301	0.607948
1.849273	1.126568	0.446376	.	0.722704
2.146408	1.000268	0.562651	0.916912	0.687684
2.009194	1.07862	0.549885	0.888275	0.676781
2.229952	1.126965	0.527515	0.983097	0.767295
2.092414	1.046207	0.479512	0.893635	0.719267
1.842644	1.24136	0.504302	0.853435	0.698265
2.346117	.	0.586529	0.94747	0.767
.	1.232934	0.627675	0.986347	0.73976
2.329177	.	0.587948	0.949762	0.768855
2.007923	1.106406	0.450758	0.881027	0.737604
1.785691	.	.	0.735284	0.612737
1.951369	1.077318	0.508169	0.874051	0.731763
1.629916	0.887399	.	.	0.615746
1.68063	0.948475	.	0.765436	0.615676
1.804932	0.963799	0.420567	.	.
1.942101	0.943825	0.435612	.	.
2.073023	1.08994	0.534284	0.833484	0.683884
1.71017	0.931281	0.507971	.	.
2.032896	1.049237	0.502759	0.98366	0.677632
2.281044	1.023272	0.511636	0.916681	0.639545
2.09499	1.10149	0.539946	0.885511	0.626337
2.075835	1.027217	0.535009	.	0.62061
2.25803	1.004036	0.525123	0.661655	0.875906
1.986254	1.037266	0.551737	0.88278	0.728293
1.960717	1.096315	0.527074	0.822236	0.737904
2.100787	1.13614	0.493042	0.964647	0.578788
1.970494	1.182297	0.437888	0.89767	0.744409
2.140521	1.080857	0.487445	0.868924	0.678185
1.83033	1.129833	0.519723	0.903867	0.723093
1.864172	1.131071	0.523644	0.921613	0.670264
1.98778	0.99389	0.507518	0.99389	0.697838
1.961255	1.047272	0.609322	0.8759	0.647405

2.033237	1.134332	0.51366	0.877502	0.642075
1.970579	1.101829	0.508537	0.847561	0.741616
1.620583	0.999934	.	0.793051	0.586168
1.942371	1.187005	0.431638	0.90644	0.733785
2.02746	1.053484	0.536681	.	0.636066
2.108706	1.166518	0.471094	0.874889	0.628125
2.015419	1.080431	0.49866	0.955766	0.644103
2.119669	1.124722	0.497473	0.90843	0.670508
2.038225	1.094205	0.47201	0.879655	0.72947
1.986185	1.112264	0.550173	0.85406	0.655441
1.841022	1.108573	0.524592	0.851225	0.692858
1.981589	1.089874	0.495397	0.871899	0.693556
1.790297	0.949954	.	0.858612	0.493245
2.101034	0.971446	0.609978	0.971446	0.609978
1.977818	1.085861	0.52354	0.891957	0.659273
2.234253	0.988227	0.53708	0.966744	0.644496
.	.	0.582445	0.931913	0.783654
2.000501	1.123877	0.494506	0.899101	0.674326
2.000501	1.123877	0.494506	0.899101	0.674326
1.980221	1.054683	0.559628	0.860966	0.710297
2.133288	1.312792	0.609511	0.961152	0.609511
2.023815	1.09286	0.607145	0.850002	0.627383
2.157144	1.150477	0.554694	0.903946	0.575238
2.173395	1.097674	0.482977	0.922046	0.680558
1.886973	1.147747	0.544693	.	0.622507
2.024356	1.144201	0.506089	0.902159	0.68212
1.999398	1.055862	0.539163	0.921071	0.673954
1.996155	0.945547	.	0.840486	0.630365
2.170131	1.043332	0.542533	0.834666	0.667733
2.122175	1.018644	0.551765	0.912535	0.636652
2.029293	0.973232	0.621212	1.056061	0.517677
2.040672	0.887249	0.687618	1.020336	0.554531
1.81875	0.927562	0.50925	.	.
2.050763	1.069963	0.512691	0.847054	0.7356
2.170461	1.040013	0.542615	0.949577	0.65566
2.04278	1.18613	0.527169	0.834684	0.593065
1.995156	1.205407	0.457223	0.893664	0.644269
.	1.36357	0.56147	0.909046	0.8021
2.064371	0.884731	0.54769	0.968991	0.653015
2.286245	0.998845	0.554914	0.910059	0.71029
2.046187	1.078099	0.528048	0.880081	0.682062
1.960798	1.132906	0.566453	0.806106	0.675386
1.976069	0.890317	0.564591	0.760027	.
2.13524	1.021202	0.557019	0.974783	0.649856
1.725826	1.076322	.	0.723734	0.556718
2.135175	0.951546	0.580211	0.905129	0.696253
2.181726	1.080272	0.593091	0.868454	0.571909

2.10882	1.19635	0.506928	0.851639	0.588036
2.181924	1.068697	0.601142	0.846052	0.601142
2.388283	.	0.685745	0.922208	0.662098
2.270794	1.167837	0.540665	0.843438	0.562292
2.037904	1.029909	0.525911	0.898431	0.657388
1.923604	1.03745	0.518725	0.907768	0.73486
2.059091	1.008096	0.536221	0.954474	0.664915
1.987495	1.141753	0.528589	0.88803	0.59202
1.891216	1.089505	0.513918	0.900383	0.698928
2.123292	1.04083	0.520415	0.936747	0.707764
2.00992	1.070501	0.50248	0.917572	0.699102
1.855711	1.184496	0.513282	0.888372	0.631731
2.218039	1.130765	0.543637	1.022037	0.717601
1.923462	1.003545	0.512226	0.972185	0.731752
2.006034	1.081906	0.518413	0.946668	0.631112
2.128349	1.170592	0.532087	0.808773	0.617221
2.030499	1.078703	0.475898	0.888343	0.719135
2.100031	1.061306	0.519362	0.92582	0.677429
2.00372	1.065808	0.468956	0.937911	0.703433
2.142067	1.071033	0.535517	0.844469	0.659097
1.94604	1.057631	0.48651	.	0.655731
2.157826	1.069193	0.524877	0.952554	0.602636
2.064733	1.098262	0.483235	0.856644	0.702888
1.985672	1.116941	0.475734	0.868732	0.703259
2.185673	1.027266	0.546418	0.896126	0.611988
2.220384	1.202708	0.578225	.	0.647612
2.091569	0.979032	0.489516	.	0.689773
2.194581	1.086426	0.43457	0.912598	0.695313
2.209266	1.008111	0.53623	.	0.600577
2.107166	1.152978	0.477094	0.874673	0.636126
2.326072	1.140231	0.570116	.	0.661334
2.090738	1.09712	0.476109	0.931517	0.641712
1.898401	1.037271	0.567563	.	0.626277
1.936209	0.958885	0.405682	.	.
1.930256	1.037968	0.509879	.	0.655559
1.832022	0.973262	0.419838	.	.
1.951421	1.05702	0.487855	.	0.75211
2.081877	1.081759	0.530674	0.857243	0.673548
2.101509	1.061368	0.467002	0.870322	0.72173
1.781582	0.99559	0.419196	.	.
2.066288	1.04358	0.500918	0.897478	0.667891
1.919816	.	.	0.790512	0.564652
2.129618	1.075147	0.45487	0.930416	0.702981
2.267454	1.175717	0.482884	.	0.776813
2.025693	1.012847	0.496868	.	0.649751
2.131141	1.044677	0.501445	0.919316	0.6477
2.108512	1.043818	0.542785	0.855931	0.709796

1.94655	1.04537	0.684897	0.775015	0.684897
1.931114	1.07864	0.643705	0.765487	0.643705
2.148935	1.092679	0.568193	0.783086	0.673819
2.138142	1.095146	0.584078	0.79963	0.591031
1.872102	1.075921	0.516442	0.968329	0.688589
2.289654	0.984337	0.449371	1.005736	0.684756
2.040337	1.083929	0.446324	0.956408	0.743873
2.013393	1.006697	0.483214	0.986563	0.744956
1.94604	1.057631	0.48651	.	0.655731
2.088905	1.012802	0.569701	0.970602	0.633001
2.007066	1.079068	0.47479	.	0.625859
2.1787	1.032016	0.504541	0.917347	0.665077
2.16814	1.02819	0.536447	0.871726	0.670559
1.935452	1.067835	0.600657	0.867616	0.622904
2.017445	1.073109	0.558017	0.794101	0.665327
2.009157	1.026903	0.535775	0.959931	0.692043
2.192349	1.085322	0.520954	0.868257	0.629486
2.076419	1.126568	0.50806	0.905672	0.618508
2.133256	1.092024	0.609502	0.99044	0.711085
2.006056	1.068443	0.588734	0.915808	0.610539
2.112411	1.023539	0.522658	0.914652	0.653323
2.024866	1.056452	0.539231	0.968414	0.616263
1.966165	1.132682	0.534284	0.876226	0.641141
2.184421	1.080466	0.469768	0.939536	0.704652
.	1.19996	0.551982	0.983967	0.887971
1.9983	1.06576	0.510677	0.888133	0.6661
1.799339	0.953649	0.449835	.	.
1.983816	1.026112	0.592864	0.889297	0.638469
2.079904	1.004897	0.514134	0.981528	0.654352
2.030447	1.003554	0.536785	0.933539	0.676816
.	1.14294	0.69108	.	0.74424
2.14093	0.999101	0.570915	0.937931	0.570915
1.81825	0.964786	0.426732	.	.
2.22578	#VALUE!	0.502596	0.909459	0.622261
2.045095	1.085152	0.459103	0.939074	0.667786
1.87095	1.053753	0.537629	.	0.645155
2.099656	1.10285	0.46659	0.890763	0.699885
2.116693	1.144742	0.453577	0.928753	0.647967
2.160275	1.182466	0.432055	0.977809	0.636713
2.046204	1.10836	0.468922	0.895214	0.724697
2.118796	1.059398	0.506669	0.990307	0.621821
1.757748	1.017643	.	0.721602	0.573581
2.100842	1.050421	0.548046	0.91341	0.639387
2.00398	1.072305	0.597678	0.808623	0.667993
1.967953	1.054261	0.602686	0.790695	0.632556
2.128474	1.12025	0.513448	0.784175	0.67215
2.121473	1.050022	0.514297	0.900019	0.685729

1.985672	1.116941	0.475734	0.868732	0.703259
2.184311	1.068918	0.487984	0.836545	0.673883
1.780255	1.007013	0.413595	.	.
2.008569	1.153435	0.556831	0.795473	0.636378
1.772147	1.090552	0.584224	0.895811	0.642647
2.156409	1.11023	0.512414	1.003477	0.576466
2.095993	1.109643	0.554822	0.883605	0.637017
2.583458	0.968797	0.507465	1.01493	0.622798
1.890591	1.027495	0.575397	0.904196	0.678147
2.020573	1.100312	0.520147	0.900255	0.640181
2.153431	1.076716	0.538358	0.848949	0.641888
2.125497	1.083186	0.572249	0.858374	0.592687
2.246012	1.100546	0.516583	0.808564	0.673804
2.155229	0.997791	0.558763	0.878056	0.678498
1.740979	.	0.461892	.	.
2.014261	1.154004	0.535038	0.818294	0.650438
2.20514	1.053567	0.539034	0.882056	0.637041
2.243211	0.964581	0.538371	0.874852	0.672963
2.120281	1.014047	0.53007	0.921861	0.645303
1.86209	0.890565	0.465523	.	.
2.068162	1.076288	0.5698	0.865251	0.654214
2.06908	1.109198	0.511937	0.895891	0.618591
2.057423	1.102948	0.509053	0.890843	0.636316
2.061434	1.061951	0.478919	0.999483	0.6455
2.11708	1.144951	0.518468	0.820908	0.669688
2.22565	1.067404	0.522346	0.885718	0.6359
2.120751	1.060375	0.508097	0.905737	0.662735
2.068431	1.095052	0.567805	0.851707	0.608362
2.008691	1.081603	0.540801	0.869145	0.676002
2.174159	1.020524	0.532447	0.843041	0.665559
2.174875	1.053806	0.470849	0.874434	0.695063
1.99379	1.139309	0.50862	0.895171	0.651034
1.989266	1.133668	0.556139	0.812818	0.684479
2.094369	1.058692	0.529346	0.897587	0.644421
2.328682	1.062602	0.497388	0.94956	0.610431
2.075933	1.027041	0.546298	.	0.633706
2.164171	1.082085	0.552084	0.839168	0.662501
2.069177	1.075972	0.537986	.	0.620753
1.976486	1.093375	0.504635	0.904137	0.65182
2.14939	1.053623	0.505739	0.906115	0.653246
2.299888	1.034949	0.528974	0.896956	0.62097
2.064733	1.098262	0.483235	0.856644	0.702888
2.326011	.	0.605732	0.944942	0.751108
.	1.081463	0.663625	1.130621	0.737361
2.345128	.	0.547927	0.876684	0.613678
2.243278	1.031908	0.605685	0.874878	0.605685
